# Remodeling of the chromatin landscape in peripheral blood cells in patients with severe Delta COVID-19

**DOI:** 10.3389/fimmu.2024.1415317

**Published:** 2024-12-06

**Authors:** Vasiliy E. Akimov, Dmitriy I. Tychinin, Olga A. Antonova, Abusaid M. Shaymardanov, Maria D. Voronina, Kseniia A. Deinichenko, Oleg D. Fateev, Vladimir S. Yudin, Sergey M. Yudin, Vladimir E. Mukhin, Svetlana V. Romanova, Aleksandra I. Nekrasova, Anastasia S. Zhdanova, Anastasia V. Tsypkina, Ivan S. Vladimirov, Antonida V. Makhotenko, Anton A. Keskinov, Sergey A. Kraevoy, Ekaterina A. Snigir, Dmitry V. Svetlichnyy, Veronika I. Skvortsova

**Affiliations:** ^1^ Federal State Budgetary Institution “Centre for Strategic Planning and Management of Biomedical Health Risks” of the Federal Medical Biological Agency (Centre for Strategic Planning of FMBA of Russia), Moscow, Russia; ^2^ The Federal Medical Biological Agency (FMBA of Russia), Moscow, Russia

**Keywords:** scATAC-seq, single cell, COVID-19, transcriptional regulatory network, PBMC (peripheral blood mononuclear cells)

## Abstract

COVID-19 is characterized by systemic pro-inflammatory shifts with the development of serious alterations in the functioning of the immune system. Investigations of the gene expression changes accompanying the infection state provide insight into the molecular and cellular processes depending on the sickness severity and virus variants. Severe Delta COVID-19 has been characterized by the appearance of a monocyte subset enriched for proinflammatory gene expression signatures and a shift in ligand–receptor interactions. We profiled the chromatin accessibility landscape of 140,000 nuclei in PBMC samples from healthy individuals or individuals with COVID-19. We investigated cis-regulatory elements and identified the core transcription factors governing gene expression in immune cells during COVID-19 infection. In severe cases, we discovered that regulome and chromatin co-accessibility modules were significantly altered across many cell types. Moreover, cases with the Delta variant were accompanied by a specific monocyte subtype discovered using scATAC-seq data. Our analysis showed that immune cells of individuals with severe Delta COVID-19 underwent significant remodeling of the chromatin accessibility landscape and development of the proinflammatory expression pattern. Using a gene regulatory network modeling approach, we investigated the core transcription factors governing the cell state and identified the most pronounced chromatin changes in CD14+ monocytes from individuals with severe Delta COVID-19. Together, our results provide novel insights into cis-regulatory module organization and its impact on gene activity in immune cells during SARS-CoV-2 infection.

## 1 Introduction

Several COVID-19 investigations have revealed a complex interplay between immune activation and the presence of specific cell subsets associated with symptom severity. The course of the disease varies significantly among COVID-19 patients. Some patients develop asymptomatic forms, while others may experience severe disease symptoms due to dysregulation of immune responses (10, 55–58). It is crucial to understand the basic and detailed molecular processes of the immune response against SARS-CoV-2, where high-throughput methods, such as single-cell transcriptome profiling, uncover gene expression characteristics of the cells to elucidate phenotypic properties.

The severity of the COVID-19 changes is characterized by quantitative and qualitative alterations in immune compartments. Severe cases are usually accompanied by pronounced immune shifts and lymphopenia (11, 26). Several investigations have reported that the fraction of neutrophils, plasma cells, and classical monocytes increases in the blood of COVID-19 patients (23, 25). Moreover, severe patients had more pronounced shifts than individuals with mild or moderate COVID-19. In addition, a decreased proportion of T cells, natural killer (NK) cells, dendritic cells (DCs), and non-classical monocytes has been identified in the PBMC of COVID-19 patients. Existing single-cell investigations of immune blood cells have identified a decreased proportion of natural killer (NK) cells in COVID-19 patients (12, 14, 15, 19, 35). However, there is a trend towards an increase in proliferating NK cells (high MKi67) with an increase in COVID-19 severity (19). In addition, multiple studies have identified an elevation of B cell levels and a decrease in T cells and DCs in severe COVID-19 patients (8, 19, 23, 35). Interestingly, proliferative T cell subsets expressing MKi67 demonstrate distinct associations with COVID-19 severity and a general trend towards lymphopenia with an increase in symptom severity. A deeper analysis found elevated levels of the activated CD4+ T cell subsets (Th1, Th2, and Th17-like) in patients with severe COVID-19 (39). Single-cell transcriptome investigations of PMBC have also identified pronounced alterations in the composition and gene expression of monocyte subtypes. In particular, CCL3, IL1RN, and TNF-high CD14 monocytes are enriched in patients with severe COVID-19 and may accompany inflammatory storms (21). Subpopulations of monocytes with high levels of CCL8, CXCL10/11, and IL6 are also elevated in PBMC with severe COVID-19 (18).

However, all genes in the genome are expressed from a single DNA sequence matrix, and precise gene regulatory programs define cell phenotypes and responses to external environmental factors. To investigate gene expression mechanisms, chromatin accessibility profiling at the single-cell level is crucial for uncovering the origins of cellular diversity. Analysis of chromatin accessibility in peripheral immune cells convalescing from COVID-19 revealed chromatin landscape changes associated with immunological memory development (27). Moreover, cell type-specific regulatory alterations revealed extensive remodeling of epigenomes in the blood immune cells during SARS-CoV-2 infection. Particularly, investigations have revealed elevated motif accessibility of the known myeloid regulators, KLF and CREB families, in individuals with mild or moderate COVID-19. Furthermore, extensive chromatin differences have been identified between seronegative and seropositive CD14+ monocytes and B cells, indicating a switch from inflammatory to adaptive immune response with the formation immunological memory (20, 23, 33).

The chromatin landscape and alterations in immune cells in response to the primary SARS-CoV-2 infection remain unknown. Here we applied single-cell transposase-accessible chromatin with sequencing (scATAC-seq) to decipher chromatin remodelingin the peripheral immune cells of healthy donors and individuals with COVID-19 (mild, convalescence, severe). In particular, here we focused on the investigation of the specific proinflammatory population of monocytes that were previously identified in individuals with the severe/critical form of Delta COVID-19 (38). We characterized the cell state based on the specific pattern of the accessible chromatin landscape, described core regulators governing the cell state, controlled the expression of specific marker genes, and delineated the transcription control from cis-elements. Our study facilitates a comprehensive understanding of immune changes together with the underlying regulatory factors leading to the formation of specific proinflammatory monocytes in severe Delta COVID-19 patients.

## 2 Materials and methods

### 2.1 Sample collection

Patients were categorized into groups based on the severity of their condition according to the World Health Organization (WHO). Healthy individuals who tested negative for COVID-19 were recruited as volunteers after they provided informed consent. Peripheral blood samples were collected from both COVID-19-positive patients and healthy individuals in EDTA tubes and processed within 4 h. Peripheral blood mononuclear cells (PBMCs) were isolated using a Ficoll Paque Plus solution and standard density gradient centrifugation. The isolated PBMCs were then suspended in freezing media (90% fetal bovine serum, 10% DMSO) and stored in a −80°C freezer.

### 2.2 Sample collection single cell RNA-seq experiment workflow

To study the 3’ gene expression of single cells, libraries were prepared using the Chromium Next GEM Single Cell 3’ Reagent Kits v3.1 protocol (10× Genomics, Pleasanton, CA, USA) platforms. Biomaterial quality control was performed using a Countess II FL cell viability counter and analyzer (Thermo Fisher Scientific, Waltham, MA, USA). Cell suspensions conforming to the protocol requirements were used for library sample preparation. In the prepared Master Mix, 31.8 μL per sample, the studied suspension of cells (13.8 μL) and water without nucleases (29.5 μL) were added. The mixture was stirred using an automatic dispenser 10 times and applied to a chip to generate an emulsion. Then, the gel particles and oil were applied to the chip. The loaded chip was processed at a chromium controller station. The resulting emulsion was incubated for 45 min at 53°C, for 5 min at 85°C and stored at 4°C. The temperature of the amplifier lid was 53°C and the volume of the mixture was 125 μL. The emulsion was cleaned after incubation as follows: 125 mL of the Recovery Agent was added to each sample; the tubes with the mixture were kept for 2 min and carefully turned over, after which 130 mL of the lower pink phase was briefly centrifuged and selected. The prepared Dynabeads Cleanup Mix was added to each sample and kept at room temperature for 10 min, after which the magnetic particles were washed with 80% alcohol and cDNA fragments were eluted using EB buffer. Amplification was performed to obtain full-sized cDNA. After cDNA amplification, magnetic particle purification was performed and quality control of the purified cDNA was carried out using an automated Tape Station 4200 (Agilent, Santa Clara, CA, USA) electrophoresis system with a set of D5000 (Agilent, Santa Clara, CA, USA) and a Qubit 4 fluorimeter (Thermo Fisher Scientific, Waltham, MA, USA). For cDNA fragmentation, 10 μL of the sample and prepared Fragmentation Mix were used. After fragmentation, the samples were cleaned bilaterally using magnetic particles. The adapters were then ligated: 50 μL of the prepared Adapter Ligation Mix was added to 50 textmu L of the sample and incubated for 15 min at 20°C. The magnetic particles were purified after ligation. The final stage of library preparation was indexing PCR. A Chromium™ i7 Sample Index Plate (10× Genomics, Pleasanton, CA, USA) was used for one-way indexing. To 30 μL of the sample, 60 μL of the prepared Sample Index PCR Mix and 10 μL of the index were added. The number of cycles in the indexing PCR was 12 for samples with cDNA concentrations of 4 ng/μL–25 ng/μL. After indexing, the samples were subjected to double-sided cleaning on magnetic particles, and quality control of the resulting libraries was carried out using a Tape Station 4200 with a set of D1000 (Agilent, Santa Clara, CA, USA) and a Qubit 4 fluorimeter. The finished libraries were sequenced with coverage of >200 million readings per sample.

### 2.3 Single cell ATAC-seq experiment workflow

To study chromatin accessibility at the single-cell level, nuclei were isolated from cell suspensions, libraries were prepared using the Chromium Next GEM Single Cell ATAC Reagent Kits v1.1 protocol and sequenced using the Illumina NovaSeq6000 and NextSeq2000 platforms. Nuclei were isolated from PBMC cell suspensions. Nuclei were isolated using the following method: cells were pelleted and resuspended in 100 μl of lysis buffer (10 mM Tris–HCl pH 7.4, 10 mM NaCl, 3 mM MgCl2, 0.1% Tween-20, 0.1% Nonidet P40 Substitute, 0.01% Digitonin, 1% BSA, nuclease-free water), incubated for 5 min and added 1 ml of wash buffer (10 mM Tris–HCl pH 7.4, 10 mM NaCl, 3 mM MgCl2, 0.1% Tween-20, 1% BSA, nuclease-free water), the nuclei were precipitated for 5 min at 500 rcf at a temperature of 4°C, then resuspended in the prepared Diluted Nuclei Buffer. Nuclear quality control was carried out using a Countess II FL counter, a cell viability analyzer, and an EVOS M7000 microscope. For sample preparation of libraries, nuclei that met the requirements of the protocol were used. The nuclei sample under study was added to the prepared Transposition Mix (10 μL per sample). The volume of the sample was calculated based on the concentration of nuclei using the formula.


(1) $$x=\frac{10,000\;*\;1.53}{n}$$

where *n* is the concentration of nuclei in the sample and *x* is the required volume of the sample. The mixture was then incubated for 60 min at 37°C. Next, 60 μl of the prepared Master Mix was added to the samples, mixed 10 times using an automatic dispenser, and applied to the chip to generate an emulsion. Subsequently, the gel particles and oil were applied to the chip. The loaded chip was processed at The Chromium Controller station. The resulting emulsion was then amplified. After amplification, the emulsion was purified: 125 μl of Recovery Agent was added to each sample, the tubes with the mixture were carefully inverted 10 times, then briefly centrifuged, and 130 μl of the lower pink phase was collected, followed by cleaning with magnetic Dynabeads MyOne SILANE and Beckman Coulter SPRIselect Reagent magnetic particles. After purification, indexing PCR was performed. We used the Single Index Kit N Set A for the one-way indexing. To 40 μl of sample, 57.5 μl of the prepared Sample Index PCR Mix and 2.5 μl of index were added. The number of cycles in indexing PCR was set to 9. After indexing, the samples were subjected to double purification on magnetic particles, and the quality of the resulting libraries was controlled using a Tape Station 4200 with a high-sensitivity D1000 kit, an Agilent Bioanalyzer 2100 with a high-sensitivity DNA chip kit, and a Qubit 4 fluorimeter. Ready-made libraries were sequenced with coverage of > 250 million reads per sample.

### 2.4 scRNA-seq feature/barcode matrix generation, QC, and filtering

The scRNA-seq data used for this study were obtained from the Single-Cell Gene Expression Analysis Revealed Immune Cell Signatures of Delta COVID-19 (38). Feature/barcode matrix of single cell RNA seq data was generated using 10× Cellranger v.6.0.1 (54). We demultiplexed the sequencing results using the mkfastq command of the Cellranger tool. The sequencing reads were aligned to the GRCh38 reference genome. Quality control and filtration we performed in R v.4.2.0 using Seurat v.4.3.0 (16). Feature/barcode matrix filtering was performed by selecting cells with 200–2,500 genes and less than 15% mitochondrial reads.

### 2.5 scRNA-seq dataset integration and annotation

The feature/barcode matrix was normalized using the Seurat (16) function, NormalizeData. We selected the top 2,000 variable genes using the FindVariableFeatures and vst approach in the Seurat package. We performed a linear transformation of the data using ScaleData, followed by the RunPCA function. To eliminate batch effects between samples, we applied the Harmony package v.0.1.1. We identified cell clusters using FindNeighbors and FindClusters (resolution = 0.5) functions from Seurat. Cell type annotation of the dataset was performed manually based on the expression of marker genes, similar to previously published scRNA-seq data (38).

### 2.6 Preprocessing and quality control of scATAC-seq data

We applied the Cell Ranger ATAC 1.2.0 pipeline (10× Genomics, Inc.) to process sequencing results (fastq files) and filter out cells based on quality metrics. According to the recommendations, we retained cells for further analysis if the following metrics were within valid values: 500–25,000 fragments fall in peak regions and at least 25% of reads in peak regions, TSS enrichment score greater than 1, and nucleosome signal score less than 3. After filtering, 112,249 cells were retained for subsequent analyses.

### 2.7 Normalization, imputation, and dimensionality reduction of scATAC-seq data

We applied the term frequency-inverse document frequency (TF-IDF) method for data normalization with default settings, as implemented in the Signac package. We selected top features with “FindTopFeatures” Signac function and performed dimensionality reduction with “RunSVD” function.

### 2.8 Cell type classification of scATAC-seq data

To identify cell types in the scATAC-seq data, we constructed a gene-activity matrix using a chromatin accessibility signal estimated with the GeneActivity function from the Signac package with default parameters. We performed cell type annotation on the scRNA-seq data based on the estimated gene activity, relying on known immune cell marker genes. We correlated the gene activity metrics obtained with scATAC-seq and scRNA-seq and identified the corresponding cell types based on the maximal correlation score.

### 2.9 scATAC-seq dataset integration with CisTopic

We applied the CisTopic R package (7) to identify cell clusters and patterns of chromatin accessibility. This method performs Latent Dirichlet Allocation (LDA) using a manually set number of topics. We identified the optimal number of topics using the runModel function and defined 13 topics as the most suitable for our data.

### 2.10 Analyses of differential open chromatin regions between cell types in scATAC-seq

For each cell type, we identified differential open chromatin regions with the Signac function “FindMarkers” using default parameters (min.pct = 0.1). Regions that were differentially accessible were considered if the adjusted p-value was ≤ 0.05 .

### 2.11 Peak annotation and GO enrichment analysis

Peak annotations have been performed with the CHIPseeker R package using the “annotatePeak” function (from −3,000 to 3,000 bp from TSS). For GO enrichment analysis, we used the seq2gene() function to assign genomic regions to the nearest genes. Next, the enrichPathway() function was used to perform enrichment analysis of the REACTOME pathways.

### 2.12 Motif enrichment analysis

We applied the PyCistarget (42) package and the run_pycistarget function for motif enrichment analysis. PyCisTarget is working on the delineation of the whole genome on segments, and the whole genome, except regions of interest, is used as the background.

### 2.13 Footprinting analysis

We used the Signac package Footprint() function with JASPAR 2020 motif collection.

### 2.14 Detection gene-regulatory networks

To build enhancer-driven Gene Regulatory Networks (eGRNs) from both scRNA-seq and scATAC-seq data, we used the SCENIC+ package (42). To generate pseudomultiome SCENIC objects from scATAC-seq and scRNA-seq, we applied the create_SCENICPLUS_object function with AnnData object from scanpy, pycistopic object with scATAC-seq, and motifs generated by pycistarget as input. We reconstructed the enhancer gene regulatory network by using the run_scenicplus function.

### 2.15 Interpretation of SEI neural network model predictions

Identification of significant motifs for determining the regulatory activity of a genomic region was carried out by decomposing the output prediction of a neural network by backpropagating the responses of all neuron models for each feature of the input signal using the DeepLIFT package. DeepLIFT compares the activation of each neuron with a reference and assigns a score for its individual contribution to the prediction, identifying the signature of the particular features of the input data that affect the prediction of the SEI neural network.

### 2.16 Identification of the network modules and graph clustering

We selected a TGF beta related network focusing on the TGFB1 gene and its neighbors. To detect modules in the network, we applied a community detection algorithm based on random walks. We used an implementation from the igraph library (1) and the function community_walktrap (steps = 4). The CellChat and Seurat R packages were used to calculate the module scores. We used genes assigned to the SPP1 and TGFB1 pathways from CellChatDB.human, provided as part of the CellChat package [Jin et al., (17)]. Selected genes were further used as inputs to the AddModuleScore() function from the Seurat package with default parameters. We used an implementation from the igraph library function community_walktrap(steps = 4). The network subgraph for the TGFB1 gene performed graph clustering with igraph.community_walktrap(n_steps = 10) from the igraph package. Algorithm behind walktrap community rallies of random walks. The general algorithm is based on the idea that random walks on a graph prefer to “stay” within the same community because there are only a few edges that lead outside a given community.

### 2.17 Gene network reconstruction with CellOracle

Monocytes were selected from the Seurat scRNA-seq object and converted to the Scanpy anndata object. The CellOrcale have been applied to the monocytes according to the description at https://morris-lab.github.io/CellOracle.documentation/. Network graphs were visualized using the NetworkX library (https://networkx.org/).

### 2.18 scATAC-seq public data workflow

For integration and data analysis, we used the SnapATAC2 package (53). Gene activity, based on scATAC-seq peaks, was obtained using the pp.make_gene_matrix function. Marker regions for each cluster were defined using the snap.tl.marker_regions function. The motif enrichment was computed using the snap.tl.motif_enrichment function.

## 3 Results

### 3.1 The immune profile of SARS-CoV-2 viral infection statements

**Figure 1:**
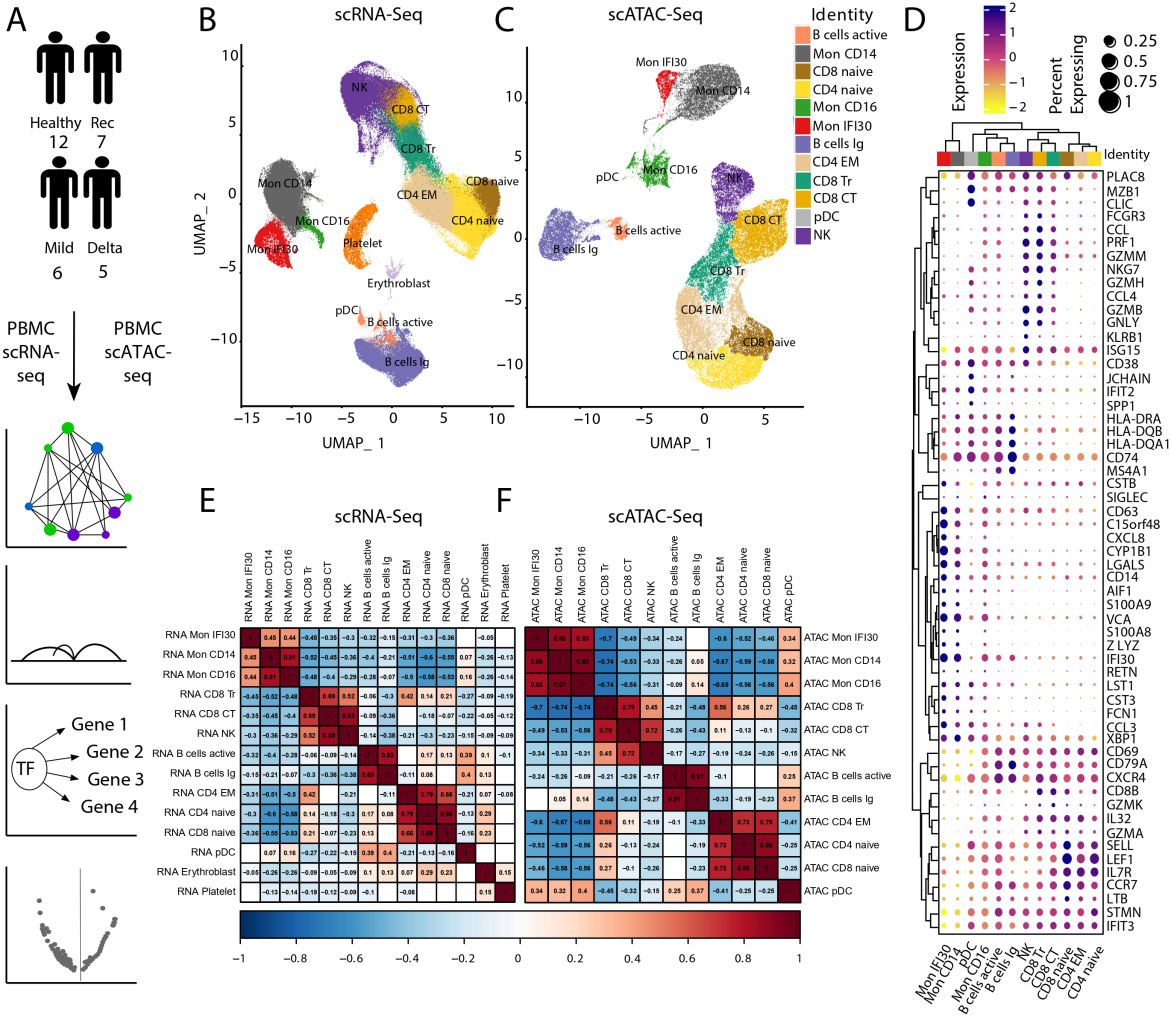
Data analysis workflow and scRNA-seq/scATAC-seq integration. **(A)** We performed scATAC-seq of the PBMC samples from 12 healthy, seven convalescence, six mild COVID-19, and five individuals with severe/critical Delta COVID-19, followed by computational analysis and integration with scRNA-seq, reconstruction of the gene regulatory networks, and analysis of differential chromatin accessibility. **(B)** UMAP of scRNA-seq and cell-type annotation. **(C)** UMAP of scATAC-seq data and cell type annotation. **(D)** Signal of the Chromatin accessibility signal for PBMC marker genes. **(E)** Pseudobulk gene expression across different cell types using scRNA-seq. The gaps are correlation values with low significance level (p-value >0.05 ). **(F)** Pseudobulk-approximated gene activity Pearson’s correlation across cell types in scATAC-seq. Gaps are correlation values with low significance level (p-value >0.05 ).

In this study, we investigated immunological shifts previously observed in a cohort of patients with severe COVID-19 caused by the Delta SARS-CoV2 variant (38). To complement single-cell transcriptome investigations, we performed scATAC-seq to profile chromatin accessibility in PBMC from mild or severe Delta COVID-19 and healthy (also convalescence after COVID-19) cohorts (**Figure 1A**). For the analysis, we used 319,943 high-quality scRNA-seq cells and 112,249 scATAC-seq cells. Furthermore, we performed a detailed investigation and coupled analysis of the transcriptome and scATAC-seq data to identify gene regulatory network properties and the role of the underlying chromatin changes. We performed data integration (see *Materials and methods*) with further annotation of cell states. First, we integrated scRNA-seq and scATAC-seq samples independently and annotated the cells according to the expression of marker genes. Part of the cell annotation has been transferred from the scRNA-seq data (**Figure 1B**, **Supplementary Figure S1**), and cell type identification for scATAC-seq (**Figure 1C**, **Supplementary Figure S2**) was performed based on the gene activity approximation via local chromatin accessibility signal. In total, we identified 12 cell types that expressed canonical lineage markers (**Figure 1D**, **Supplementary Figure S3**). To estimate the similarity between gene expression and chromatin accessibility, we performed a correlation analysis. Row and column-based hierarchical clustering indicated corresponding grouping of myeloid and lymphoid cell types based on gene expression (**Figure 1E**) and chromatin accessibility measurements (**Figure 1F**). Both methods demonstrate high-resolution power and precisely demonstrate the common knowledge similarity of the cell types. We also evaluated the agreement between direct gene expression from scRNA-seq and approximate gene activity via chromatin accessibility signals. Our results demonstrated high concordance between both data types (**Supplementary Figure S4**).

Previously, we showed that the proinflammatory subtype of monocytes (Mon IFI30) accompanies severe Delta COVID-19, and this population yields high expression of IFI30, C15orf48, CXCL8, CSTB, and SPP1 genes (38). We applied an unsupervised approach that uses genomic intervals to discover coaccessible cis-elements and stable cell states from the scATAC-seq data. Based on the clustering results, we identified a specific group of cells with high chromatin accessibility near Mon IFI30 marker genes (**Figure 1D**, **Supplementary Figure S5**). This indicated an agreement between the gene expression patterns and chromatin accessibility profiles. Applying region-based cell clustering and topic modeling allowed us to avoid gene activity estimation by scATAC-seq, which imperfectly corresponds to transcriptome profiles due to possible distant or condition-specific cis-regulations. Moreover, separation of Mon IFI30 cells into a separate cluster purely based on the open chromatin profile provides additional confidence in the transcriptome and chromatin accessibility interconnections governing cell type-specific regulatory networks. Using scATAC-seq, we gained new insights into the gene regulatory mechanisms during COVID-19 in immune cells.

### 3.2 Immunological shifts of the PBMC composition depending on the COVID-19 severity and virus variant

**Figure 2:**
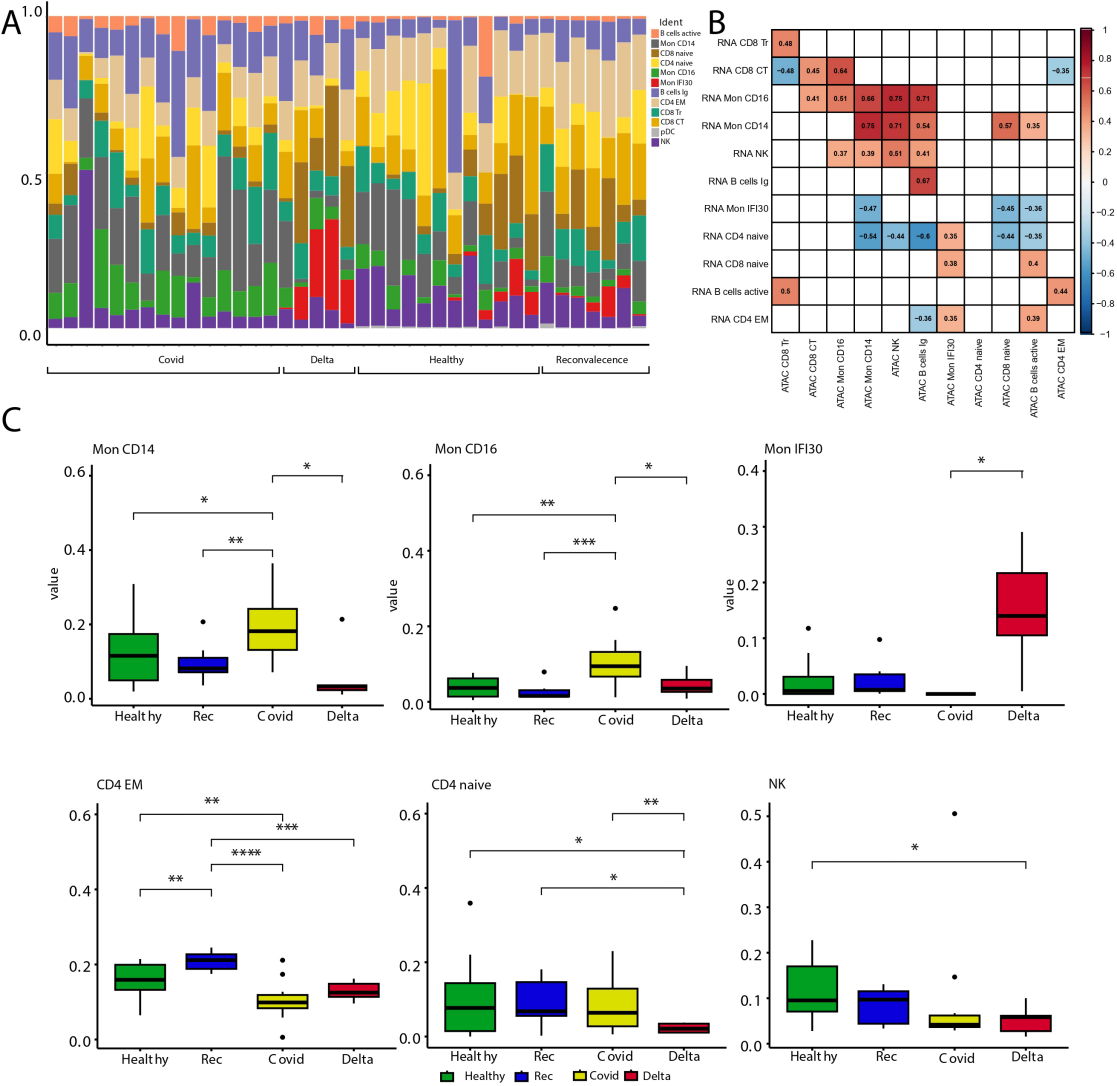
Analysis of cell type abundance across the study cohorts. **(A)** Barplot with fractions of cell types across cohorts. **(B)** Heatmap showing Pearson correlation of scRNA-seq pseudobulk gene expression and approximated gene activity from scATAC-seq across cell types. Gaps are correlation values with low significance level (p-value >0.05). **(C)** Boxplots with cell fractions across the study cohorts. Pairwise comparisons were performed using Wilcoxon rank-sum test. Significant changes (adjusted p-values <0.05 ) are shown with stars. * stands for p-value < 0.05; ** stands for p-value < 0.005; *** stands for p-value < 0.0005; **** stands for p-value < 0.00005.

Previously, we characterized the immune landscape changes in PBMC depending on the severity of COVID-19 symptoms and the SARS-CoV2 virus variant. Our analysis showed that both lymphoid and myeloid compartments underwent significant compositional changes. Moreover, the Mon IFI30 cell state almost exclusively accompanies severe/critical COVID-19 caused by the Delta variant but not the Wuhan-like virus (38). Here, we checked the agreement between PBMC composition profiled using scRNA-seq and scATAC-seq methods. We identified enrichment of Mon IFI30 cells for severe/critical Delta COVID-19 samples based on scATAC-seq data (**Figure 2A**). However, we also traced Mon IFI30 in other cohorts, but at much lower levels. We hypothesize that this indicates clustering artifacts, which are also caused by the initially low number of samples with severe/critical Delta COVID-19 (only four individuals in our collection).

Next, we compared the cell type fractions recovered using scRNA-seq and scATAC-seq. Correlation analysis indicated clear similarities and agreement in the results between experimental approaches that point to the biologically relevant clustering and cell type assignment (**Figure 2B**). We also investigated the cell state composition that significantly changed between the study groups (**Figure 2C**, **Supplementary Figure S6**). To this end, we performed cell fraction analysis and calculated all pairwise differences across cohorts. Similar to our previous study, scATAC-seq showed that both myeloid and lymphoid compartments underwent significant compositional shifts. Severe/critical Delta COVID-19 is accompanied by depletion of classical CD14 monocytes and enrichment of Mon IFI30 (**Figure 2C**). In addition, only mild/moderate COVID-19 samples were enriched for CD16 monocytes, and severe Delta cases were accompanied by the depletion of classical Mon CD14. Our results indicate serious shifts in the immune response and exhaustion of compensatory mechanisms, especially in severe/critical COVID-19 cases caused by the Delta variant. Similar to previous results, we identified depletion of NK and CD4 Naive cells with an increase in COVID-19 severity.

It should be noted that annotation of scATAC-seq yields fewer cell types due to noise caused by the high dimensionality of the data and dropout rate. However, with the current scATAC-seq data we managed to recover the main trends in the composition of blood immune cells across the study cohorts. Additional high-resolution properties of scATAC-seq will help elucidate gene regulatory differences across cell phenotypes.

### 3.3 Analysis of the cis-regulatory topics across cell types

**Figure 3:**
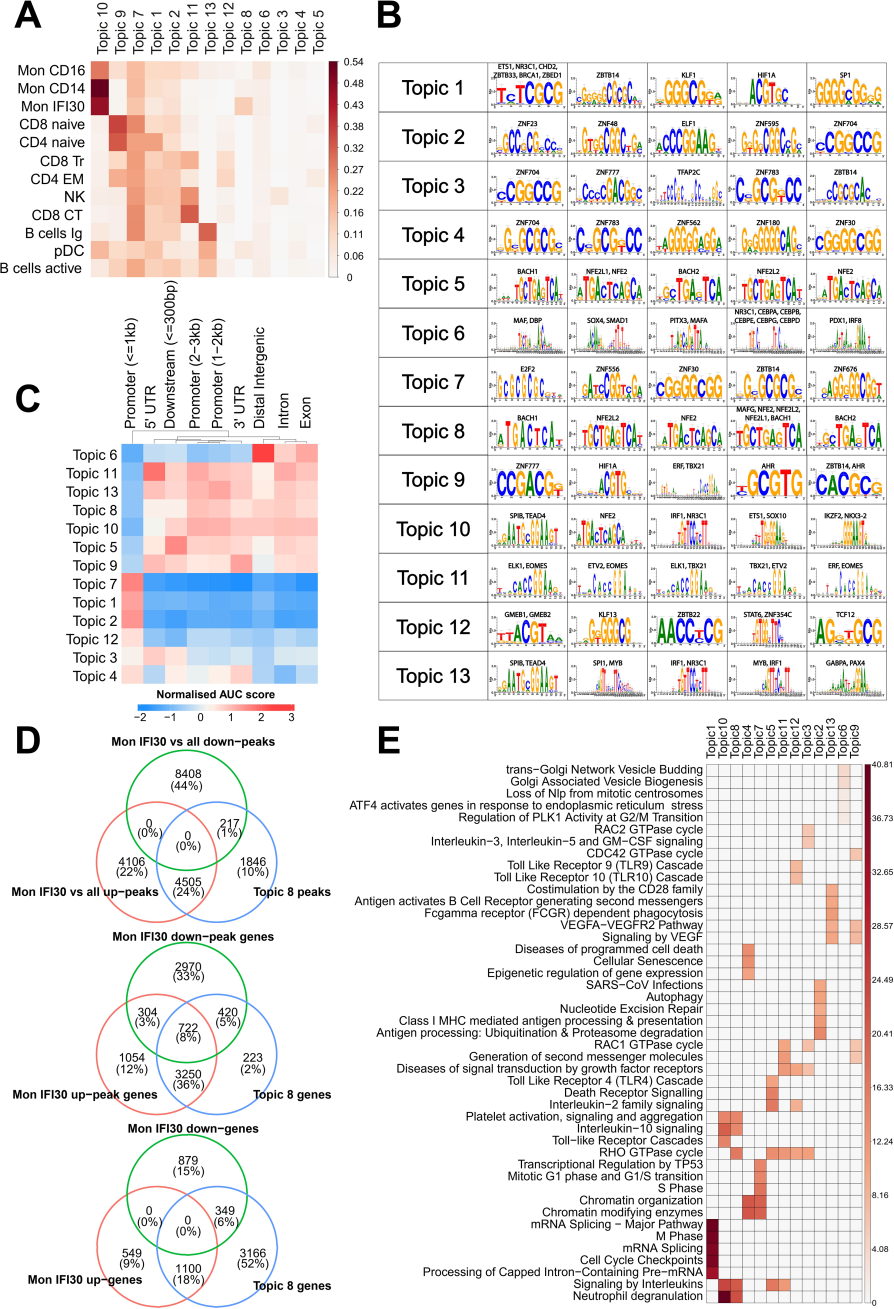
Analysis of cis-regulatory topics. **(A)** Jaccard metric for overlap of cis-regulatory topics and cell type-specific marker peaks. **(B)** Top-enriched *de novo* discovered motifs in cis-regulatory topics. **(C)** Normalized enrichment of cis-regulatory topics in genomic segments, as estimated using cisTopic. **(D)** Intersection of the Mon IFI30 specific up/downregulated marker peaks with topic 8. We found that topic 8 strongly overlapped with upregulated but not downregulated marker peaks. We also assigned scATAC-seq peaks and topic 8 loci to the closest genes and found a high overlap with marker genes of the Mon IFI30 cell state. **(E)** Enrichment of the Reactome pathways based on the assignment of topic cis-regulatory regions to genes. Colorbars show the −log(p.adjusted).

We applied cisTopic to identify groups of cis-elements representing various regulatory programs governing cell states. Our analysis shows that this approach precisely recovers distinct cell clusters and allows the characterization of regulatory loci, together with operating transcription factors. To this end, we identified the optimal number (**Supplementary Figure S7**) of regulatory themes (topics) and their averaged representations in the cell clusters (**Figure 3A**). We quantified the overlap of the 13 topics with marker peaks (see *Materials and methods*) for each cell type and identified a common and specific set of themes governing the regulatory landscape of the cell subtypes. Topic 8 was mostly specific to Mon IFI30. However, topic 10 was common for myeloid cells, and topic 7 was the second most pronounced across monocytes and other cell types (**Supplementary Figure S8**). Identification of both shared and cell type-specific topics reflects distinct regulatory programs, as measured by chromatin accessibility.

Next, we identified TFs that might operate in the regulatory regions for each topic (**Figure 3B**). Motif enrichment analysis showed that SPIB, NFE2L2, TEAD4, ETS1, IRF1, IKZF2, and NKX3-2 are the main regulatory proteins for the common across monocytes in topic 10. Interestingly, TEAD4 has been shown to be a marker of severe COVID-19 (44). In addition, we identified higher expression of the NFE2L2 factor in the Mon IFI30 cluster (**Supplementary Figure S9**). We further investigated the distribution of regulatory regions around transcription start sites (TSS), promoters, exons, and introns (**Figure 3C**). In particular, myeloid topics 10 and 8 mostly operate within a distance of more than 1 kB from TSS. Interestingly, topic 8 was enriched for BACH-binding sites (**Figure 3B**), and BACH1 was overexpressed in Mon IFI30 (**Supplementary Figure S9**).

Next, we investigated the association between the Mon IFI30 marker genes and the peaks from topic 8. First, we investigated the overlap between topic loci and up/down peaks obtained in the differential analysis of Mon IFI30 compared to all other cell types. We identified that 4,505 upregulated (marker) Mon IFI30 peaks were common to topic 8 (**Figure 3D**). The overlap of the genes associated with topic 8 and the up/downregulated peaks was the highest in the upregulated group. Finally, we used up/down Mon IFI30 genes and identified the enrichment of the topic 8 regions in the vicinity of the marker genes. This highlights that regulatory elements from topic 8 indeed colocalized with the Mon IFI30 specific gene regulatory landscape. In addition, the association was high for the upregulated peaks and genes, indicating the role of the topic 8 regions in marker gene expression.

To understand the main biological processes associated with the regulatory themes discovered with cisTopic, we performed a GO enrichment analysis using ChIPseeker (**Figure 3E**). The most pronounced in monocytes, topics 10 and 8, were associated with previously reported gene alterations in COVID-19 (38), characterized by neutrophil degranulation and platelet activation. Both topics (10 and 8) have similar associated GO categories with a difference in the RHO GTPase cycle that is specific for topic 8. We hypothesized that regulatory regions from topic 8 complement topic 10 and bring about additional activation of the core processes elevated during COVID-19, contributing to the development of severe respiratory forms with exacerbations. Together, we identified groups of open chromatin regions that are specific for the Mon IFI30 cell type that accompanies severe/critical Delta COVID-19. These sets of regions showed high agreement with marker genes and peaks of the Mon IFI30 cell type. Moreover, myeloid-related regulatory topics also showed enrichment for platelet activation and neutrophil degranulation, similar to previous reports (24, 37, 38, 43, 49).

### 3.4 Transcriptional regulatory landscape changes in the immune cells during COVID-19

**Figure 4:**
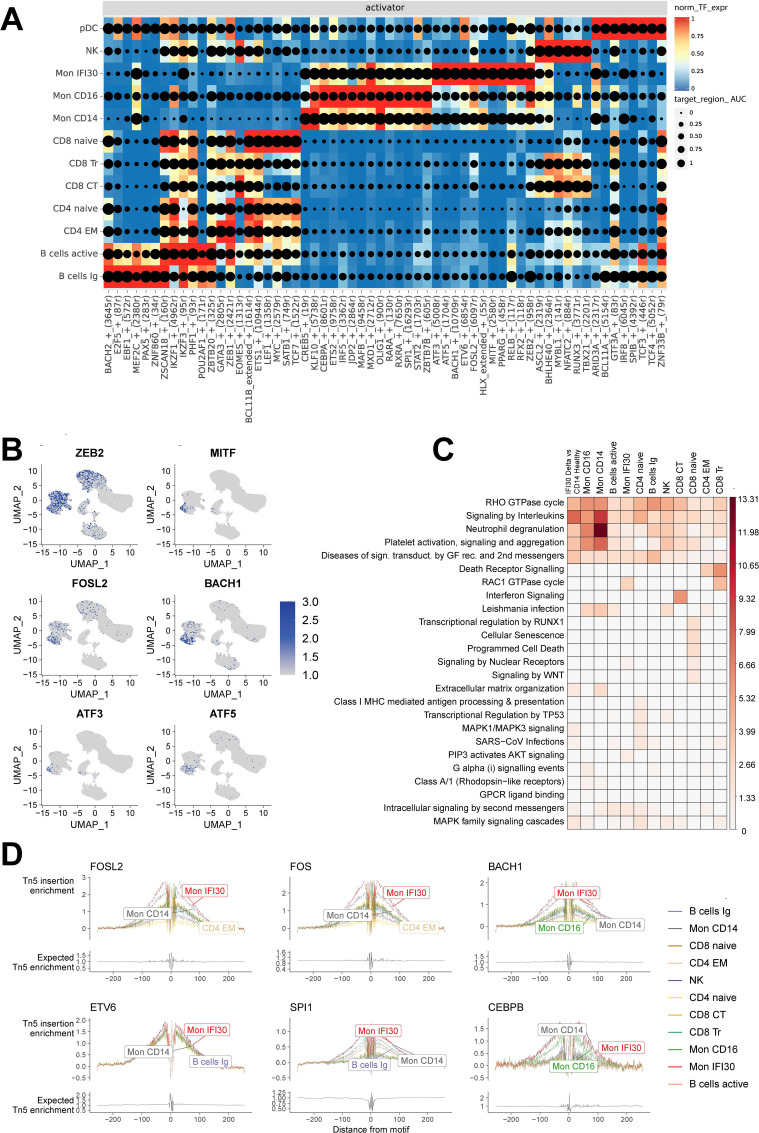
Joint scRNA-seq/scATAC-seq analysis revealed the core transcriptional regulators in each cell state. **(A)** Heatmap/dotplot with TF expression of enhancer-driven regulons (color scale) considering the cell-type specificity of the regulons (dot size scale). **(B)** Pseudoexpression (estimated gene activity) of the core transcriptional regulators (ZEB2, MITF, FOSL2, BACH1, ATF3, and ATF5) in the Mon IFI30 cell state across all cells. **(C)** Enrichment of the Reactome Pathways for upregulated peaks between Delta COVID-19 and healthy individuals. The peaks were assigned to the closest gene. **(D)** Footprint profiles for Mon IFI30 regulators (FOSL2, FOS, BACH1, ETV6, SPI1, and CEBPB) across cell types.

The primary focus of our study was the Mon IFI30 cell subpopulation. Here, we performed scATAC-seq on four out of nine PBMCs samples from individuals with severe/critical Delta COVID-19 that have been previously studied using scRNA-seq (38). To obtain a joint representation of the transcriptome and open chromatin we applied the SCENIC+ package to gain insights into the core transcription factors that govern cell states and control regulatory regions. First, we identified that the SCENIC+ pseudo-multiome approach captures biological representation and allows splitting cell types into separate clusters with specific activity of the core regulons (**Supplementary Figures S10, S11**). In addition, we reconstructed gene regulatory networks with TF-gene and peak-gene association and identified core TFs operating in these regions. We detected the main activators governing Mon IFI30 cell state: ZEB2, MITF, FOSL2, BACH1, and ATF3/ATF5 (**Figure 4A**). Moreover, ETS3, IRF5, MAFB, and SPI1 were common for both Mon IFI30 and Mon CD14. We also confirmed that core Mon IFI30 regulators were expressed in myeloid cells (**Figure 4B**). The majority of these regulators were previously identified to be elevated during COVID-19 (13, 38), and our analysis suggests their potential role in the establishment of the proinflammatory Mon IFI30 cell state. In addition to activators, we identified regulators with predicted repressor function (**Supplementary Figure S12**). For several of these TFs, we also identified a complementary negative regulatory role (MITF, FOSL2, and BACH1).

Previously, we identified that Mon IFI30 gene expression data indicated the enrichment of the platelet overactivation and neutrophil degradation signals (38). We performed GO enrichment analysis based on the upregulated peaks in the severe/critical Delta COVID-19 cohort, in contrast to the healthy individuals (**Figure 4C**). We identified activation of neutrophils and Toll-like receptor cascades in the myeloid compartment. Overall, GO enrichment performed on the upregulated peaks recapitulated results similar to those obtained with expression data and topic-based analysis. In addition, cellular senescence, programmed cell death, and WNT signaling are overactivated in CD8+ Naive cells. CD8 cytotoxic cells demonstrated signatures of interferon activation that can lead to the high regulatory activity of EOMES in CD8 CT cells (**Figure 4A**).

Using SCENIC+, we effectively identified cell states from the reconstructed gene regulatory network, coupled with motif enrichment analysis of open chromatin. We applied a footprinting approach to investigate motif-binding sites, together with the chromatin accessibility profile. Bound to the DNA, TFs prevent DNA cleavage in nucleosome-free regions, which is reflected in the aggregation plot of the read distribution as a drop of the read coverage centered at the binding site. We investigated footprinting of the top up/down regulators (BACH1, FOSL2, CEBPB, ETV6, CEBPB, SPI1, and FOS) of the Mon IFI30 state and confirmed their active regulatory profiles (**Figure 4D**, **Supplementary Figure S13**). We further analyzed the distribution of the BACH1 and FOSL2 target regulatory regions (**Supplementary Figure S14**) and identified that more than half of the cis-elements fell in promoters or were concentrated near TSS (UTRs, 1st intron, 1st exon), indicating proximal binding preferences of the TFs.

Altogether, coupled analysis of gene expression and chromatin accessibility profile recapitulates core biological properties and allows identification of the transcriptional regulators governing cell state and phenotype formation.

### 3.5 Cis-elements regulating Mon IFI30 state

**Figure 5:**
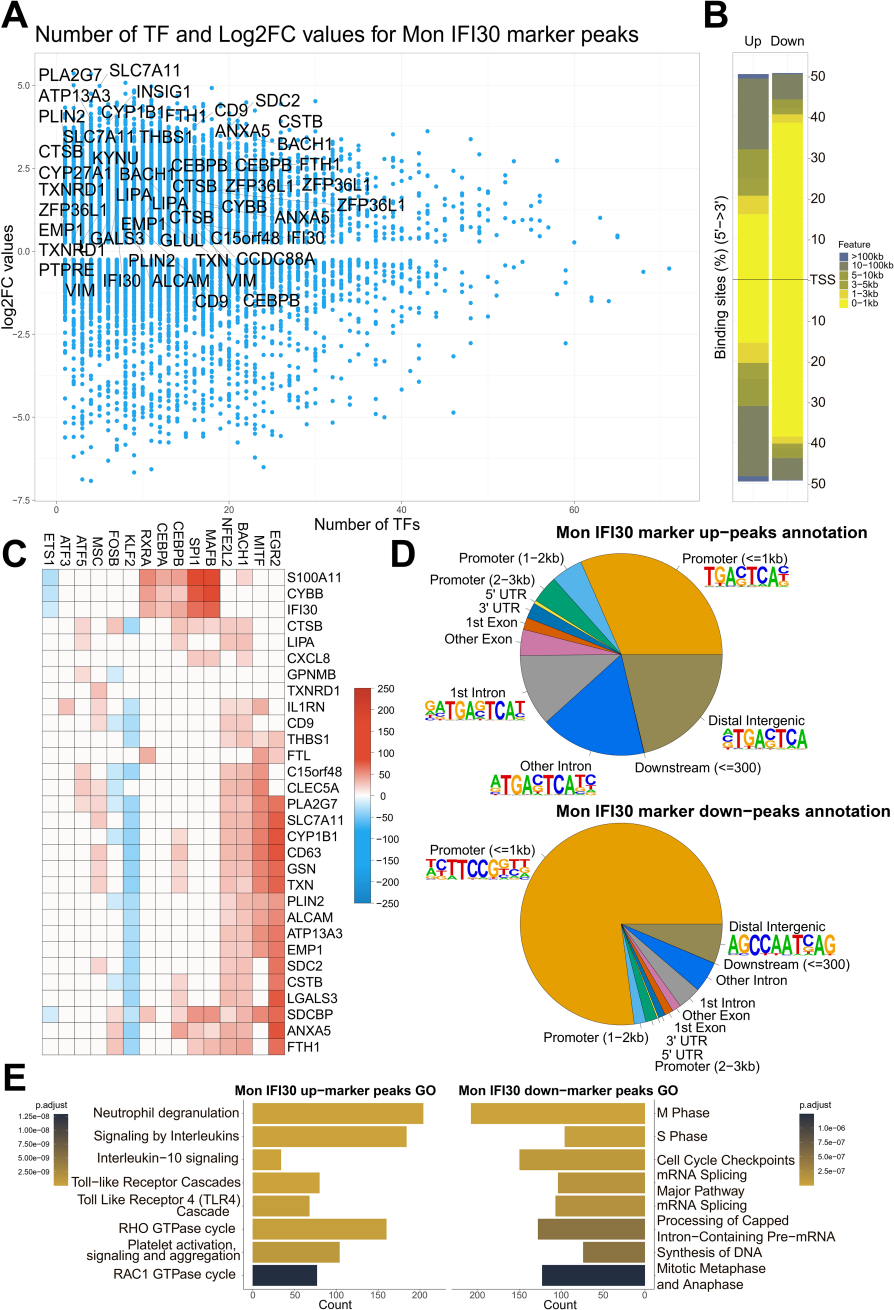
Mon IFI30 cis-regulatory elements. **(A)** Scatterplot of the number of TFs operating in the Mon IFI30 marker peaks (as estimated using SCENIC+). The highlighted genes represent Mon IFI30 expression markers. **(B)** Barplot showing the distribution of the Mon IFI30 marker peaks around TSS. **(C)** Heatmap showing the contribution of transcription factors in the regulation of Mon IFI30 marker genes. **(D)** Distribution of Mon IFI30 marker peaks by UTRs, exons, introns, promoters, and distal intergenic locations. The top enriched motifs within each segment are highlighted with logos. **(E)** Enrichment of the Reactome Pathways for up/down marker peaks in Mon IFI30.

The Mon IFI30 cell state is characterized by the expression of the proinflammatory genes (SPP1, CSTB, IFI30, LGALS, and CXCL8), and we identified an association of regulatory topic 8 with specific marker genes and differentially accessible regulatory regions linking transcriptome and epigenome changes. Next, we took a locus-centric approach, focusing on the core transcriptional regulators operating in cis-regulatory elements. First, we identified that Mon IFI30 marker peaks with a high fold change are usually regulated with fewer TFs (**Figure 5A**). We hypothesized that loci with a low number of regulators are more sensitive to perturbation of the operating TFs. Mon IFI30 specific genes were in the vicinity of the upregulated marker peaks with low numbers of predicted regulatory TFs. Moreover, peaks under the control of many TFs tended to be more proximal (**Supplementary Figure S15**). This, in turn, explains why promoters usually harbor regulatory information as hubs that aggregate multiple input signals. However, because promoters are usually under the control of multiple TFs, the effect of a single perturbed regulator can be compensated for, ultimately leading to changes in chromatin accessibility. In addition, the distribution of the marker peaks around the TSS showed the prevalence of the downregulated peaks to be proximal to the TSS (**Figure 5B**).

To investigate the most important upstream regulators of the Mon IFI30 cell state, we took a closer look at the TFs that govern the activity of maker genes. The reconstructed gene regulatory networks predicted that the most expression-activating input cane from EGR2, MITF, SPI1, MAFB, BACH1, and NFE2L2, which are specifically or highly expressed in Mon IFI30 (**Figure 5C**, **Supplementary Figure S9**). In addition, several TFs have demonstrated a repressor activity. For instance, KLF2, FOS, and ETS1 were predicted to be negative regulators. Moreover, both KLF2 and FOS were downregulated in myeloid cells coming from individuals with severe Delta COVID-19 (**Supplementary Figure S9**). Interestingly, the expression of KLF2 was decreased in the PBMC of individuals with the Delta COVID-19 variant (**Supplementary Figure S9**). This is in line with the normal anti-inflammatory role of KLF2, and its low expression can be partially responsible for the development of COVID-19 exacerbation (2, 4). Our analysis suggests that EGR2, MITF, BACH1, and NFE2L2 as the main regulators of the marker genes (CD9, CD63, IL1RN, CXCL8, and C15orf48). Both CD9 and CD63 are involved in exosome formation (31), and COVID-19 plasma exosomes stimulate pro-inflammatory responses that affect CD4/CD8 T cells, and CD14+ monocytes. Plasma exosomes have been hypothesized to serve as cargo for SARS-CoV-2 dsRNA (30). Interestingly, both ATF5 and ATF3 were exclusively expressed in Mon IFI30 and contributed to the regulation of core marker cell type genes (IL1RN, C15orf48, and CLEC5A). Particularly, CLEC5A can be stimulated by the SARS-CoV-2 spike protein and contributes to the cytokine storm. scRNA-seq data are less noisy and demonstrate that Mon IFI30 cells are specific to patients with severe or critical Delta COVID-19. Thus, ATF3/ATF5 polarity may reflect the heterogeneity within the population.

We further investigated the distribution of the up/downregulated peaks around TSS and core gene elements, such as UTRs, exons, introns, and identified regulatory motifs enriched across up/down marker peaks (**Figure 5D**). For the upregulated peaks, we identified the domination of the consensus sequence for TFs governing topic 8 activity. For the downregulated peaks, the STAT motif was overrepresented in both proximal and distal peaks.

We also investigated how Mon IFI30 cell states differed across cohorts. To this end, we performed separate monocyte clustering using scVI (32). The clustering highlights biological differences because we identified grouping first on the cell type and only after based on cohort assignment (**Supplementary Figure S20**). We also investigated how general Mon IFI30 marker peaks (identified based on marker peak discovery using all cohorts) overlap with marker peaks of Mon IFI30 found separately for each cohort. Our analysis showed similarity across cell states as well as individual differences because the Mon IFI30 state from each study cohort partially overlapped with general marker peaks. Analysis of the approximated gene activity yielded qualitatively similar results (**Supplementary Figure S21**). Moreover, we identified that the gene module activity of the Mon IFI30 markers discovered with scRNA-seq was the highest for Delta COVID-19 as well as for the scATAC-seq data (**Supplementary Figure S24**).

We further analyzed the core Reactome pathways associated with Mon IFI30 specific regulatory elements (**Figure 5E**). Our analysis showed that open chromatin Mon IFI30 marker peaks were enriched for neutrophil activation, cytokine signaling, TLR activation, and platelet aggregation. Region-based and transcriptome enrichments highlight the convergence of regulatory and expression signals to the phenotype associated with COVID-19 related exacerbations leading to cytokine storm and thrombosis complications. In addition, downregulated Mon IFI30 peaks are enriched for basic processes characterized by inhibition of the cell cycle, nucleic acid metabolism, and processing, which can indicate stress-induced changes leading to myeloid blood cell dysfunction (6).

Taken together, our results suggest that Mon IFI30 open chromatin alterations were strictly coupled with previously revealed transcriptome changes. Joint analysis of scRNA-seq and scATAC-seq revealed core TFs driving the formation of the Mon IFI30 cell state that accompanies severe/critical Delta COVID-19 and represents one of the core changes in peripheral blood immune cells during a cytokine storm caused by SARS-CoV-2 specific variant.

### 3.6 TGF-beta regulatory network in COVID-19

**Figure 6:**
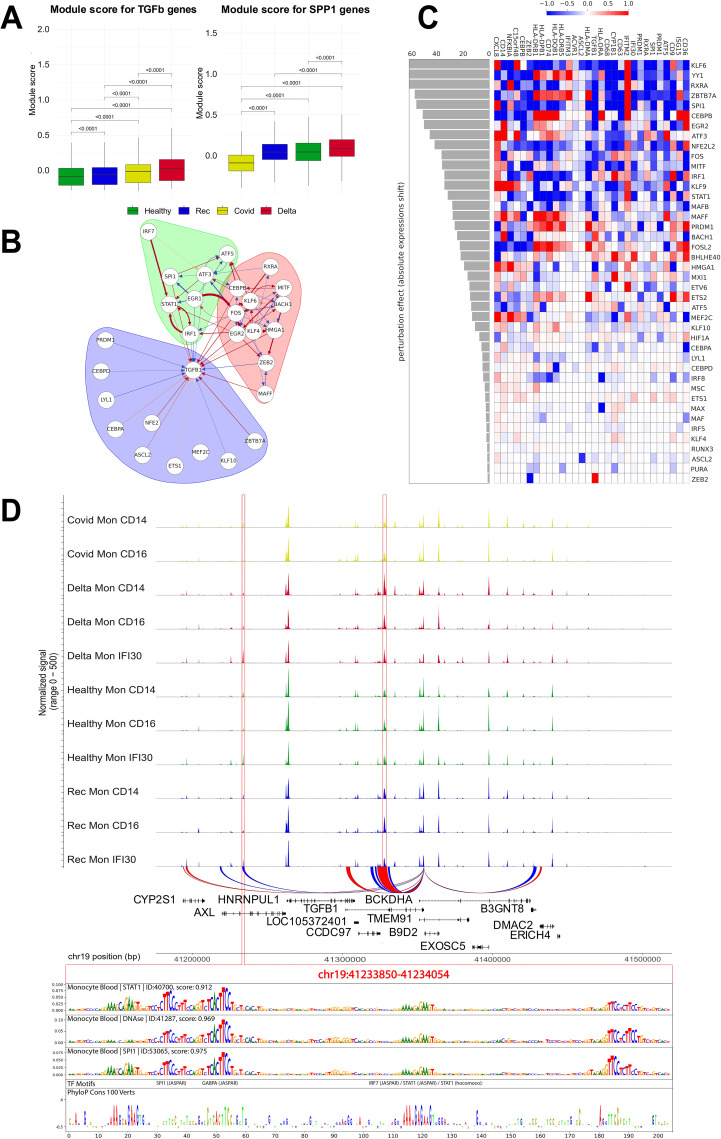
TGF beta gene regulatory network and *in silico* perturbation of Mon IFI30 cell state regulators. **(A)** Boxplot with aggregated scores for TGF beta and SPP1 modules (obtained from the CellChat database). Significant differences are highlighted with p-values. Module score have been calculated for all monocytes (Mon CD14, Mon CD16, and Mon IFI30). **(B)** Gene regulatory subnetwork centered on TGFB1. Modules of the network were estimated using a community detection algorithm based on random walks. **(C)** Heatmap barplot with *in silico* perturbation of transcription factors in Mon IFI30. Barplot shows the absolute perturbation effect on the expression of Mon IFI30 marker genes in a single computational knockdown of a TF. **(D)** Chromatin accessibility signals across monocyte subtypes and cohorts. We identified two (highlighted with red frame) loci with significantly elevated chromatin openness in Mon IFI30, and distally located regions present exclusively in Mon IFI30 (left red frame). The DeepLift method highlights important regulatory nucleotides that overlap strongly with conserved sequences across mammalian positions.

Previously, it was shown that Delta COVID-19 is characterized by a specific pattern of the ligand–receptor interactions (38). High activity of the SPP1 and TGF beta ligand–receptor interacting pathways is common for the Delta lineage, in contrast to the Claudin and Resitin pathways for the Wuhan-like cases. Here, we performed a deeper investigation of the TGF beta and SPP1 gene modules as the most pronounced for the severe Delta COVID-19 (38), relying on the CellChat ligand–receptor interaction database (17). First, we found that Mon IFI30 cells had elevated module scores for TGF beta and SPP1 pathways (**Figure 6A**). Moreover, TGF beta demonstrated a gradual increase with the increase in COVID-19 severity.

Next, we investigated TGFB1 gene regulation in Mon IFI30. To decode complex regulatory connections between multiple regulators and TGFB1, we applied a gene network reconstruction approach using CellOracle (46). Focusing on TGFB1, we splitted TGFB1 centered network into regulatory modules by applying graph clustering (**Figure 6B**). Three interconnected components are identified. The purple module contained TGFB1 and its direct regulators (CEBPD, NFE2, ETS1, and KLF10). The green and red modules have strong interconnections mediated by FOS and EGR1. The green module has a strong regulatory input orchestrated by IRF7, STAT1, and IRF1, whereas the red module mainly contains TFs related to inflammation and is represented by FOS, KLF4/KLF6, EGR2, ZEB2, and MITF. Interestingly, many of the mentioned TFs have been highlighted as high-impact regulators that control the Mon IFI30 cell state (**Figure 4A**). We identified positive and negative interconnections between regulators both within and between defined communities that together point to complex combinatorial regulation of the proinflammatory Mon IF30 phenotype.

Furthermore, we focused on the possible expression output of the TFs. We applied CellOracle to simulate the TFs perturbation effect on the expression of Mon IFI30 marker genes (**Figure 6C**). First, we selected TFs based on their high predicted activity with respect to TGFB1 to capture which other Mon IFI30 specific genes they may affect. We perturbed 42 TFs and identified the top regulators based on the perturbation score. First, we estimated the effect of each perturbed TF on the global expression shift of the markers. Our analysis highlights KLF6, YY1, and RXRA as the top activators, and BACH1 and ZEB2 as the most valuable repressors. Next, we focused on TGFB1 and its TFs. Our analysis indicates that RXRA, SPI1, ZBTB7A, CEBPB, EGR2, MITF, FOS, and MAFF are the most important TGFB1 activators.

To decipher the regulatory relationships between cis-regulatory elements and operating TFs, we focused on ATAC-seq peaks in the vicinity of TGFB1. We reconstructed peak–gene associations with SCENIC+ and selected different open regions targeting TGFB1 activity in Mon IFI30. We found a distally located enhancer nearly 96 kb away from the TGFB1 gene (**Figure 6D**). Next, we investigated the architecture of the TGFB1 regulatory region in Mon IFI30 to decode its binding motif composition. To this end, we applied the Sei model (29) and identified high scoring (prediction value > 0.9 ) classes of regulatory assays in monocytes (DNA-seq, SPI1, STAT1). We used DeepLift (5) to highlight the importance of each nucleotide for classification and complemented this with a sequence conservation regulatory profile. Our analysis showed that important regulatory motifs overlap with highly conserved positions, indicating the functionality of the predicted regulatory elements within the TGFB1 enhancer. Our analysis indicated that several TFs could operate as TGFB1 enhancers in Mon IFI30. Using deep learning model, we highlighted the relevance of the classification binding sites and identified high scoring matches to GABPA, ETS-family, PRDM1, and STAT-family TFs. We also examined the expression of candidate TFs across cohorts and monocyte subtypes. Indeed, almost all subtypes of monocytes from individuals with Delta COVID-19 were characterized by higher expression of STAT1, PRDM1, EGR2, ATF5, NFE2L2, BACH1, MAFF, MSC, PURA, GABPA, ETS2, ATF3, and ZEB2 (**Supplementary Figure S16**). Moreover, each study cohort was characterized by a particular expression pattern of several TFs. For example, individuals with moderate COVID-19 demonstrated lower levels of FOSL2, MAX, MAFB, IRF1, RUNX3, and HIF1A in CD14 and CD16 monocytes from other cohorts.

The discovered architecture of the TGFB1 enhancer indicates a complex structure in which several TFs can experience cooperativity or compete for the binding site. We also want to highlight that a differential increase in DNA accessibility occurred in the severe/critical Delta COVID-19 group. However, all the training regulatory data in the Sei model do not rely on SARS-CoV2 infection and the associated regulatory shift. Furthermore, Deep Neural Networks learn regulatory DNA signals by relying on the collection of cis-elements, which together represent joint activity profiles of TFs expressed in the cell and their binding preference to DNA sequences. However, we showed that the expression pattern of the many regulatory factors significantly changed in the case of severe/critical Delta COVID-19, which might make transfer of the predictions obtained for far regulatory data biased towards the composition of the Sei training set.

Altogether, we investigated the Mon IFI30 cell state in terms of the underlying gene regulatory network and simulated the perturbation response. Our analysis indicated that core transcription factors are involved in the regulation of core marker genes. We delineated complex interactions between regulators and the target gene TGFB1, predicted core regulators having an effect on TGFB1 expression, and deciphered the sequence composition of the distal cis-element regulating the expression of the target gene via the combined activity of several TFs with pronounced binding sites in the area.

### 3.7 Gene regulatory network governing Mon IFI30 cell state

**Figure 7:**
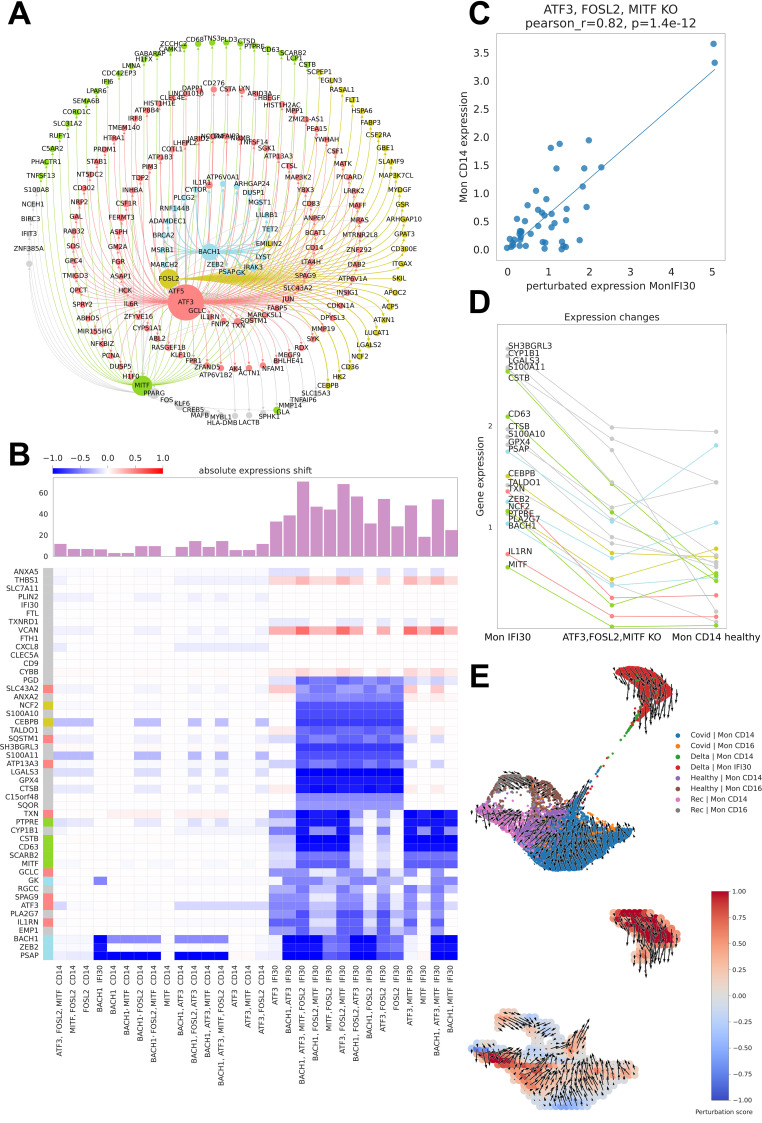
Master regulators of Mon IFI30 cell state. **(A)** Graph of the CellOracle gene network containing Mon IFI30 marker genes and core transcriptional regulators. The nodes are colored according to the regulatory TF. The size of a node reflects the number of incoming and outcoming connections for the regulators. **(B)** Heatmap with the TF perturbation response. The barplot on top of the heatmap shows an absolute expression shift across all genes for every perturbation. *In silico* perturbations for single or multiple TFs (mentioned at the bottom of the plot). **(C)** Scatterplot of in CD14 monocyte expression values predicted in Mon IFI30 after joint knockdown of FOSL2, MITF, and ATF3. **(D)** Lineplot showing changes in the expression of the Mon IFI30 marker genes after joint *in silico* knockdown of FOSL2, MITF, and ATF3. **(E)** UMAP plot of monocytes across the study conditions with the direction of the vector fields upon joint perturbation of the FOSL2, MITF, and ATF3 TFs.

The gene regulatory network governs the phenotype and properties of the cell type and defines the response to perturbations by environmental factors. To estimate core regulators and their relationship with target genes, we applied cell type-specific gene regulatory network reconstruction from single-cell data using CellOracle (46). To further explore the core regulators of the Mon IFI30 cell state, we selected a subnetwork of marker genes and controlling TFs (**Figure 7A**). Interestingly, we found that only five TFs (ATF3, ATF5, BACH1, MITF, and FOSL2) regulated most of the Mon IFI30 marker genes.

Mon IFI30 represents a highly pro-inflammatory cell state with a significantly altered pattern of gene expression accompanied by a specific set of marker genes. Next, we examined the impact of perturbation of individual TFs or their combinations with respect to gene expression changes. To this end, we simulated the regulatory perturbation that switched off one or multiple TFs at a time (**Figure 7B**). Our analysis yielded a high regulatory role of the TFs, particularly in Mon IFI30 cells, and their regulatory impact on Mon CD14 was insignificant. According to our analysis, *in silico* knockout of FOSL2, BACH1, ATF3, and MITF led to high expression shifts. In particular, BACH1 perturbation strongly changed the expression of ZEB2 and PSAP. Furthermore, ATF3 and MITF had highly overlapping targetomes. However, the effects of MITF on CD63 was higher than that of ATF3, which is a stronger regulator of ILR1N. Previous studies have found that both CD63 and ILR1N correlate with COVID-19 severity (22, 41). Moreover, our analysis points to a broad targetome role of FOSL2, which regulates the expression of most Mon IFI30 marker genes. We further investigated the combined effect of joint perturbation on BACH1, MITF, FOSL2, and ATF3. The predicted expression shifts exceeded the effect of each TF. We found that the triple in silico knockdown of ATF3, MITF, and FOSL2 shifted the expression of the Mon IFI30 marker genes towards Mon CD14 (**Figures 7C, D**). Ultimately, our predictions indicated that triple downregulation of ATF3, MITF, and FOSL2 dramatically decreased the level of the marker genes in Mon IFI30, making it close to the classical Mon CD14 cells (**Figure 7E**).

In addition, *in silico* knockdown of BACH1, MITF, FOSL2, and ATF3 did not alter the expression of several Mon IFI30 marker genes. For example, CXCL8, IFI30, and CD9 levels remained unchanged. We further investigated the direction of trajectories within the myeloid compartment when ATF3, MITF, and FOSL2 were computationally switched off. Complex TF–gene interactions recovered with CellOracle and complemented with regulatory network modeling indicated a global gene expression shift from Mon IFI30 towards classical Mon CD14 cells (**Figure 7D**). In addition, the perturbation score indicates the global influence of gene expression dynamics and demonstrates that the main changes involve Mon IFI30 cells (**Figure 7E**).

Overall, computational reconstruction and modeling of the gene regulatory network have identified the core governing TFs in myeloid cells in healthy conditions and in the case of COVID-19. Importantly, these results predict a novel association between regulatory factors controlling transcription and target expression markers of the proinflammatory Mon IFI30 cell state that accompanies the severe form of Delta COVID-19.

## 4 Discussion

Our study reports single-cell transcriptome and chromatin accessibility analyses of peripheral blood mononuclear cells derived from samples of healthy people or individuals with COVID-19. Here, we characterized the cis-regulatory level Mon IFI30 cell subpopulation that we previously identified using scRNA-seq in the PBMC of patients with severe/critical Delta COVID-19. Specifically, based on scATAC-seq data, we identified a separate cell cluster that corresponded to the Mon IFI30 cell state with highly proinflammatory properties. Our joint analysis of both scRNA-seq and scATAC-seq data allowed us to perform computational reconstruction of the gene regulatory network, together with associated open regulatory regions and controlling TFs. Several other studies have shown important regulatory roles for various TFs and their expression changes during COVID-19 of different severities (40, 50).

Complementary to other investigations, our study followed the previously characterized subtype of Mon IFI30 cell state. Using scATAC-seq, we identified a separate cluster of Mon IFI30 cells based on the open chromatin pattern. Thus, we confirmed coupled gene expression and chromatin accessibility changes that accompany the monocyte phenotype switch for severe Delta COVID-19. We performed a detailed investigation of the cell type-specific open cis-regulatory regions for Mon IFI30 using an unsupervised LDA approach to group regions into regulatory topics and to cluster cells according to the contribution of each topic to a cell. In this way, we identified a Mon IFI30 state as a separate cluster of cells characterized by the presence of a set of open regulatory elements associated with marker genes of the query monocytes. Our in-depth analysis of the cis-regulatory elements coupled with the identification of the binding sites for transcription factors allowed us to identify potential master regulators governing the expression of the marker genes. To strengthen this analysis, we reconstructed the gene regulatory network and pruned it with motif enrichment to focus on direct cis-regulatory effects (3). We discovered a high impact of BACH1, NFE2L2, MITF, EGR2, FOSL2, CEBPA, and CEBPB on the regulation of the core Mon IFI30 marker genes. GO/KEGG-based investigation of the Mon IFI30 marker peaks revealed extra neutrophil activation and elevation of the proinflammatory cytokine signaling, confirming the previously identified immunological shifts uncovered by scRNA-seq. Previous studies of blood monocytes using scATAC-seq highlighted the altered activity of CEBPD, CEBPB, and ATF4 in patients with severe COVID-19 (52). However, SPI1, RUNX1/2, IRF4, STAT2, and BCL11 motifs are overrepresented in chromatin-accessible genomic areas of convalescent-specific monocytes (52). Our investigations of open chromatin have identified that the Mon IFI30 cell subtype accompanies severe Delta COVID-19 but not a mild form of convalescence. Using scATAC-seq data, we significantly complemented current knowledge not only about gene expression activity but also shed light on the cis-regulatory elements and controlling TFs orchestrating gene activity levels.

We uncovered TF-gene regulatory interactions and their structure in the Mon IFI30 cell state and performed reconstruction of the gene regulatory networks. This analysis revealed that ATF3, FOSL2, MITF, and BACH1 are the core master regulators of the pro-inflammatory Mon IFI30 state. Here, we identified the major role of the combined perturbation of ATF3, FOSL2, and MITF in shifting Mon IFI30 expression towards classical Mon CD14. Furthermore, ATF3 was shown to be upregulated during COVID-19 (36), and demonstrated elevation upon CXCL8 stimulation, which is dramatically overexpressed during Delta COVID-19. In agreement with previous studies, we also highlighted the elevation of ATF3, CEBPB, FOSL2, and MITF with COVID-19 severity (28, 34). We hypothesize that overactivation of TFs significantly contributes to the development of severe COVID-19 and the formation of the proinflammatory cell subtypes (47). We also investigated the regulation of the TGFB1 expression by Mon IFI30 and identified distally located enhancers under the control of GABPA and STAT TFs according to cis-element deciphering using deep learning modeling. Joint analysis of the single-cell transcriptome and accessible chromatin data allowed us to identify TF–gene regulatory interactions complemented by associations between the location of the cis-regulatory elements and regulators orchestrating enhancer activity obtained by analysis of the TF binding sites.

In summary, our investigation significantly improved the understanding of the core immune shifts during COVID-19 of different severities, depending on the virus variant. Moreover, we investigated changes in gene expression in the context of alterations in chromatin accessibility during COVID-19. Coupled analysis of the scRNA-seq and scATAC-seq data identified TF-gene regulatory interactions, together with the location of the cis-regulatory elements and particular regulators orchestrating enhancer activity. Despite significant progress in understanding the role of transcriptional and regulatory signals governing the formation of proinflammatory Mon IFI30 cells, our study has certain limitations. Expansion of the sample size by individuals with COVID-19 caused by other SARS-CoV-2 variants would be beneficial for understanding the general and variant-specific properties leading to immune shift and uncoverong links between the individual properties of the infectious agent and its interactions with the host. We compared the identified Mon IFI30 chromatin landscape with PBMC samples from infants with acute Omicron and pre-Omicron variants (51). Our analysis revealed that PBMC from infants with acute Omicron and pre-Omicron COVID-19 demonstrated elevated chromatin accessibility of Mon IFI30 marker genes (**Supplementary Figures S25–S28**). Moreover, we identified that more than half of the Mon IFI30 marker peaks were shared with monocytes from infants affected with COVID-19 (**Supplementary Figure S29**), and BACH1, NFE2L2, FOS, and FOSL2 are the master regulators of the cell state (**Supplementary Figure S30**). This comparison brings additional contributions to the field and raises additional questions about the similarities and differences brought by different SARS-CoV2 variants that should be further investigated. In addition, several previous scRNA-seq studies have investigated the immune shift during infections caused by SARS-CoV-2 (10, 15, 25, 27, 45), HIV (21, 48), influenza (15), and sepsis (9, 21) and identified both common similarities and specific cohort differences in gene expression levels. Our investigation additionally contributes to the aforementioned studies and provides new information based on the reconstructed regulatory interactions and identified transcriptional master regulators.

## Data Availability

The scRNA-seq data presented in the study are deposited in the NCBI BioProject repository, accession number PRJNA1164162. The scATAC-seq data presented in the study are deposited in the NCBI GEO repository, accession number GSE282769.
