# Supplementary material for: Remodeling of the chromatin landscape in peripheral blood cells in patients with severe Delta COVID-19
Source: Front Immunol. 2024 Dec 6;15:1415317. doi: 10.3389/fimmu.2024.1415317 (PMC11662282; doi:10.3389/fimmu.2024.1415317)
Supplement: Supplementary file 1 [file DataSheet1.pdf]

Supplement

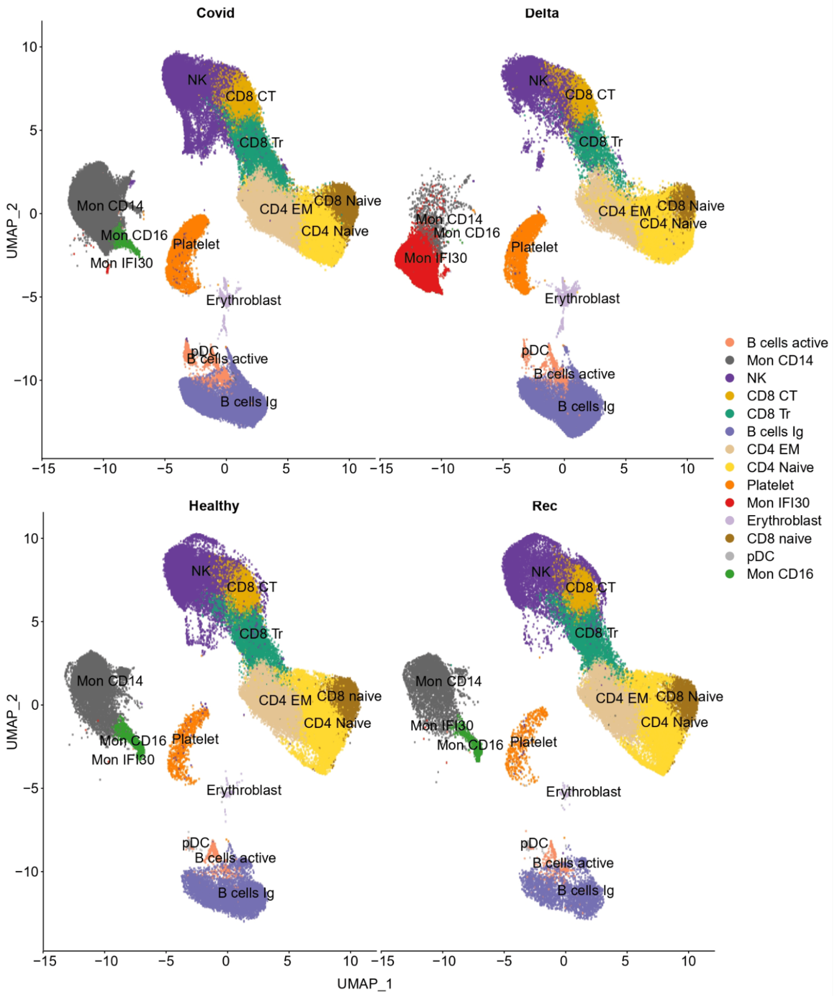

Supplementary figure 1 UMAP for scRNA-seq data splitted by study cohort.

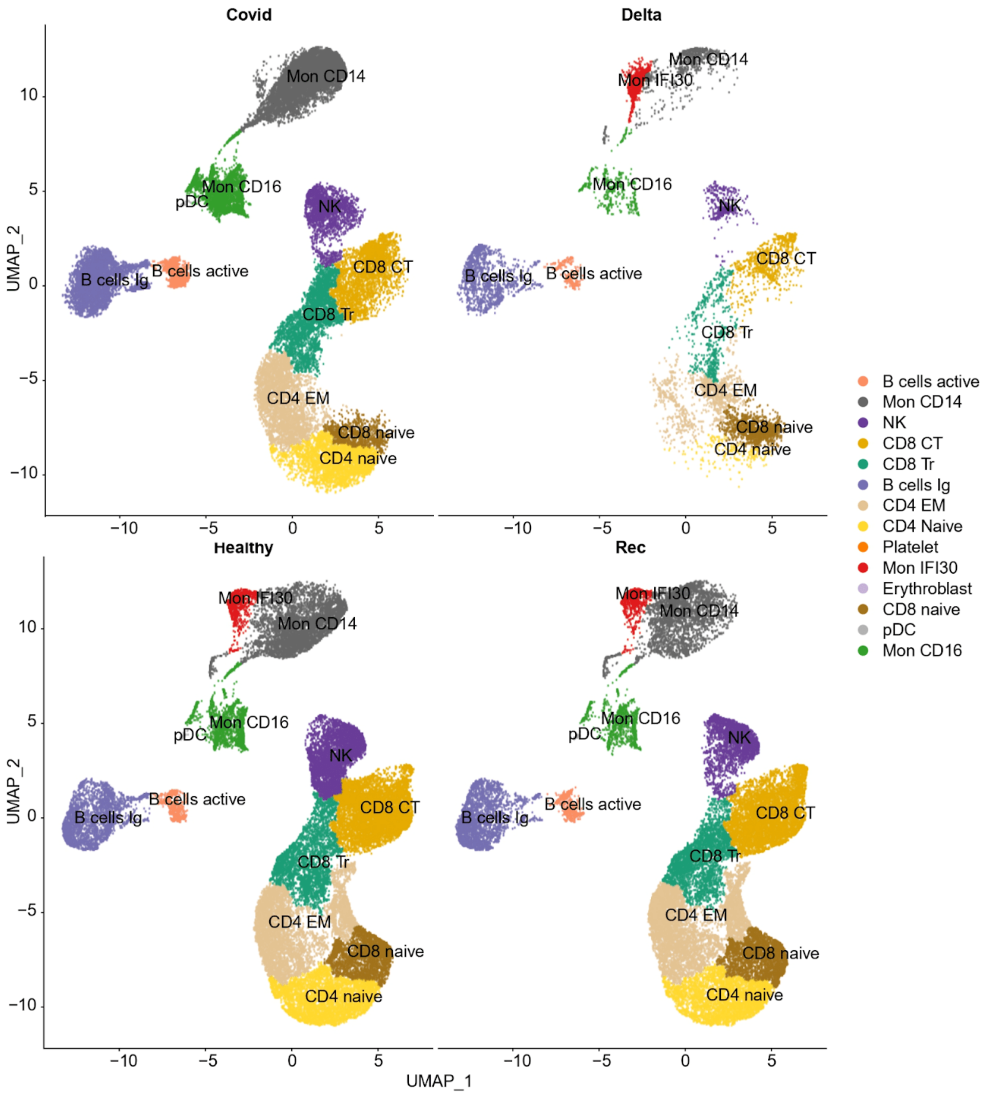

Supplementary figure 2 UMAP for scATAC-seq data splitted by study cohort.

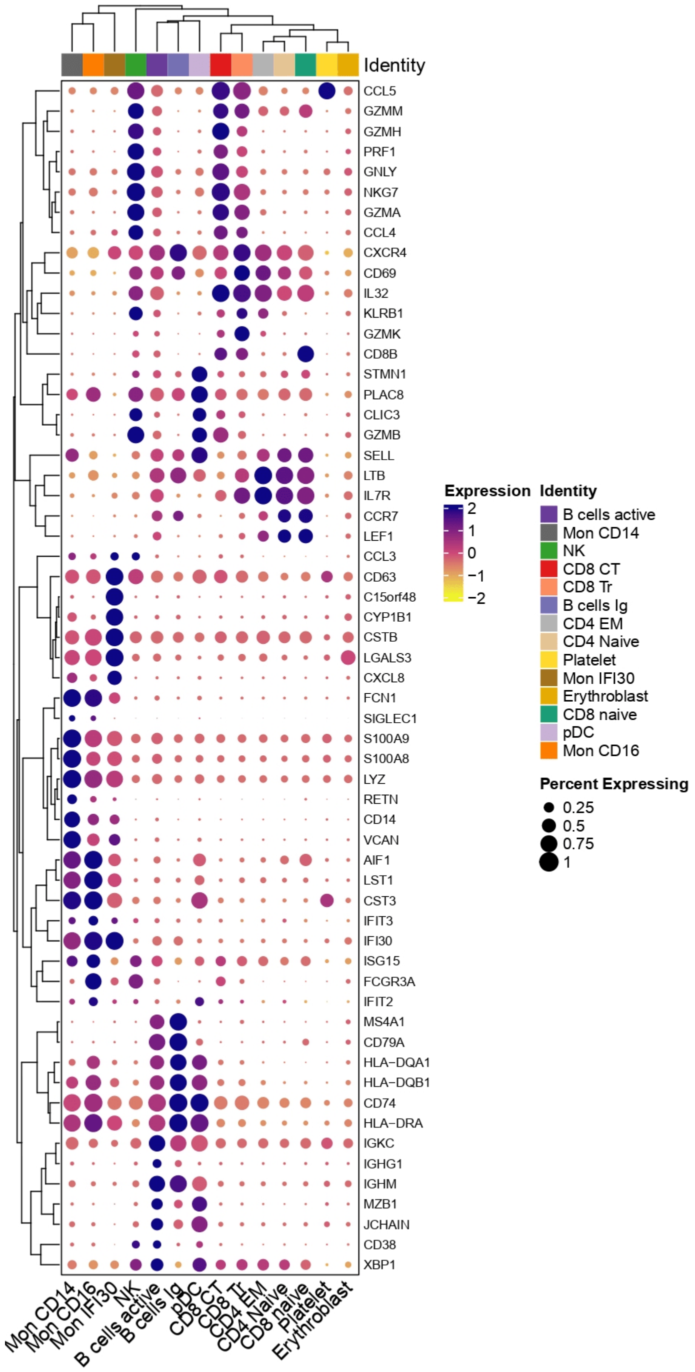

Supplementary figure 3 Dotplot depicting the expression level of marker genes across cell states.

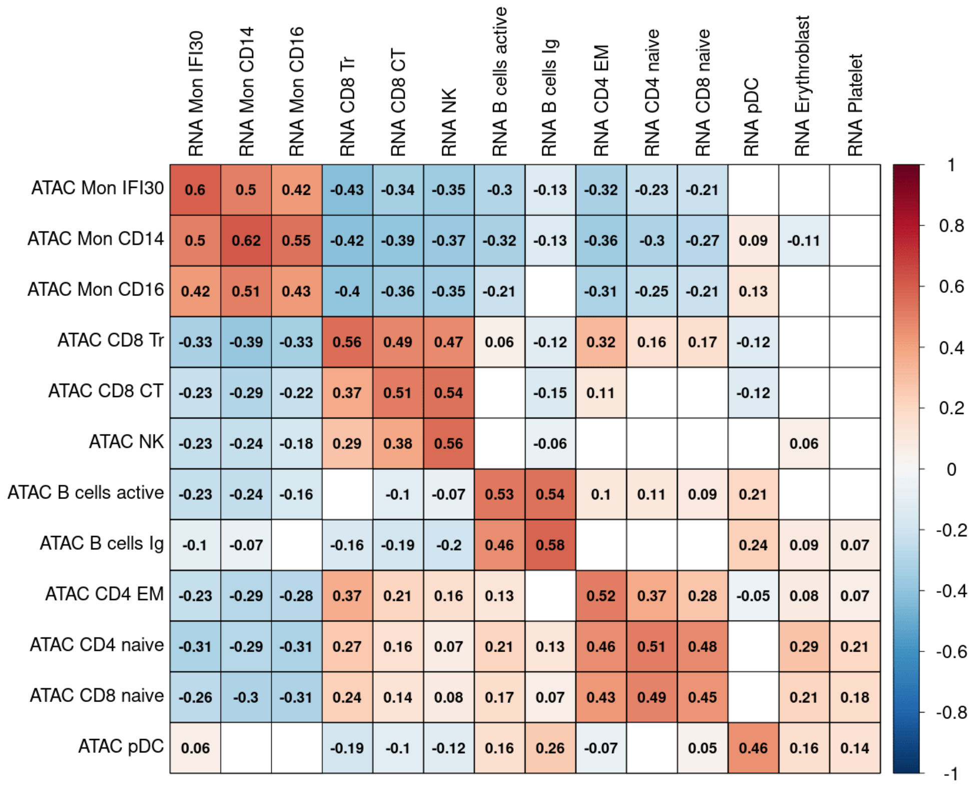

Supplementary figure 4 Heatmap with Pearson correlation of annotated cell types between pseudobulk scRNA-seq data expression and pseudobulk scATAC-seq data approximated expression.

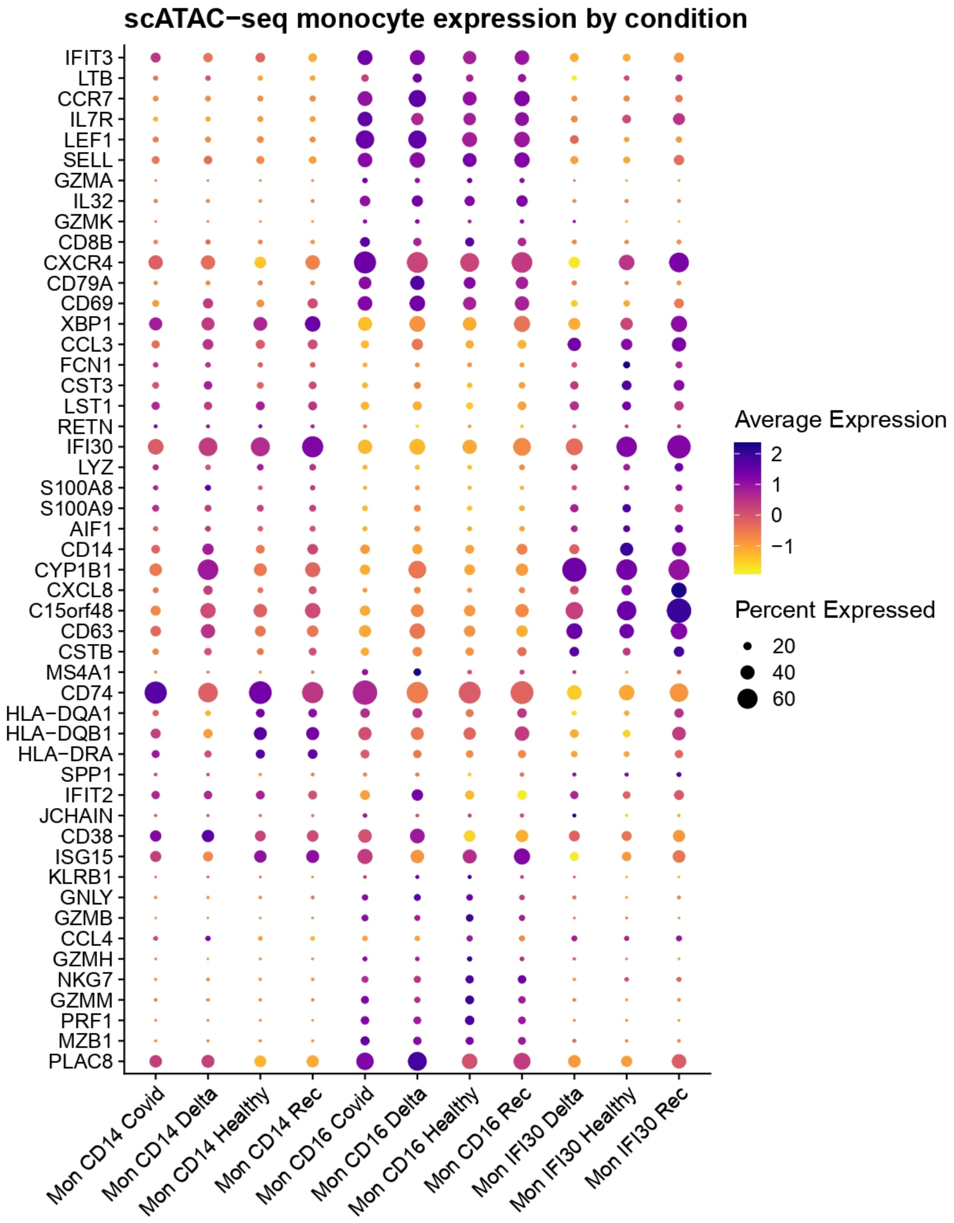

Supplementary figure 5 Dotplot depicting marker gene expression levels between monocyte subtypes across cohorts.

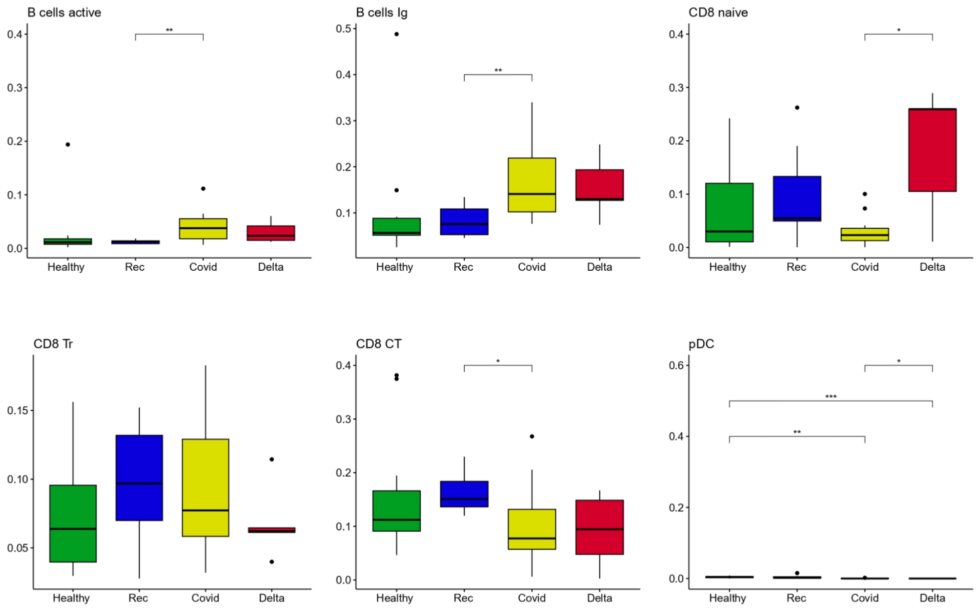

Supplementary figure 6 Boxplots with cell fractions across study cohorts. Significant changes (p-adjusted < 0.05) are indicated with asterisks.

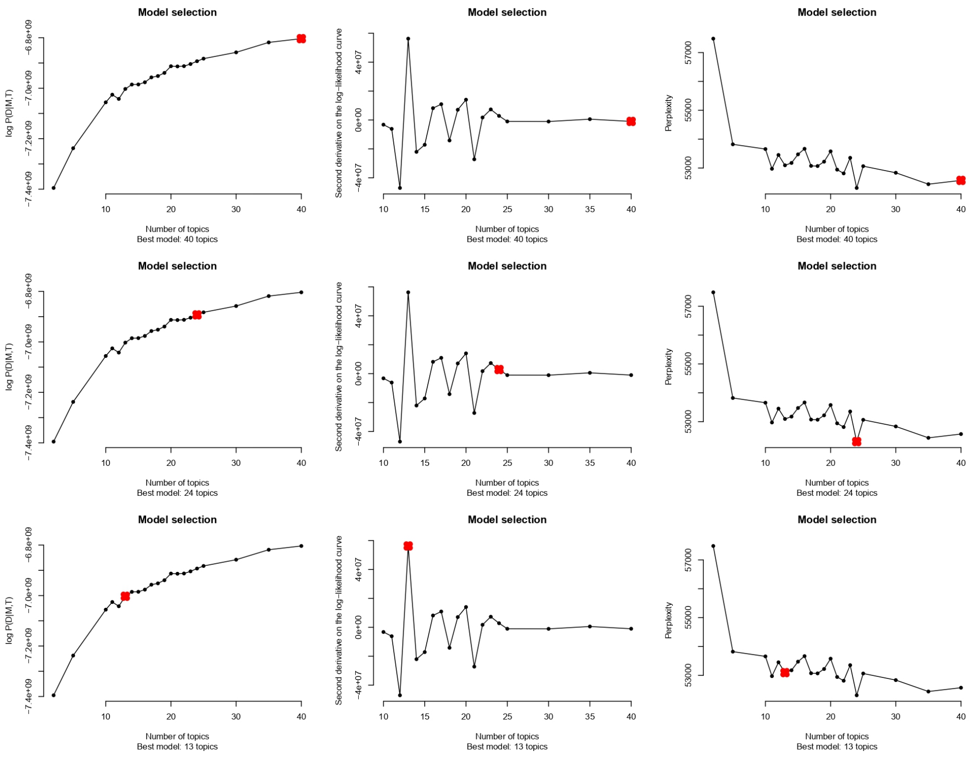

Supplementary figure 7 Statistical evaluation of the resulting CisTopic models based on the number of cis regulatory topics.

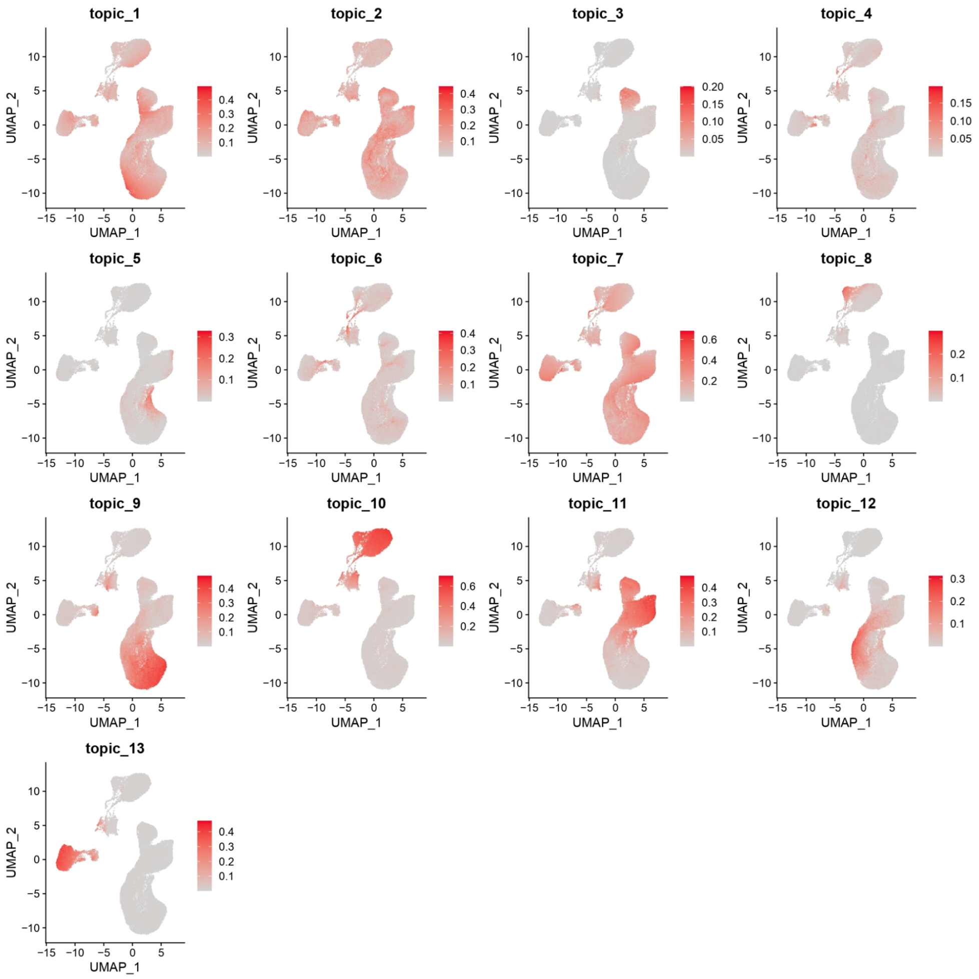

Supplementary figure 8 UMAP with highlighted probability assignments of cells to cis regulatory topics.

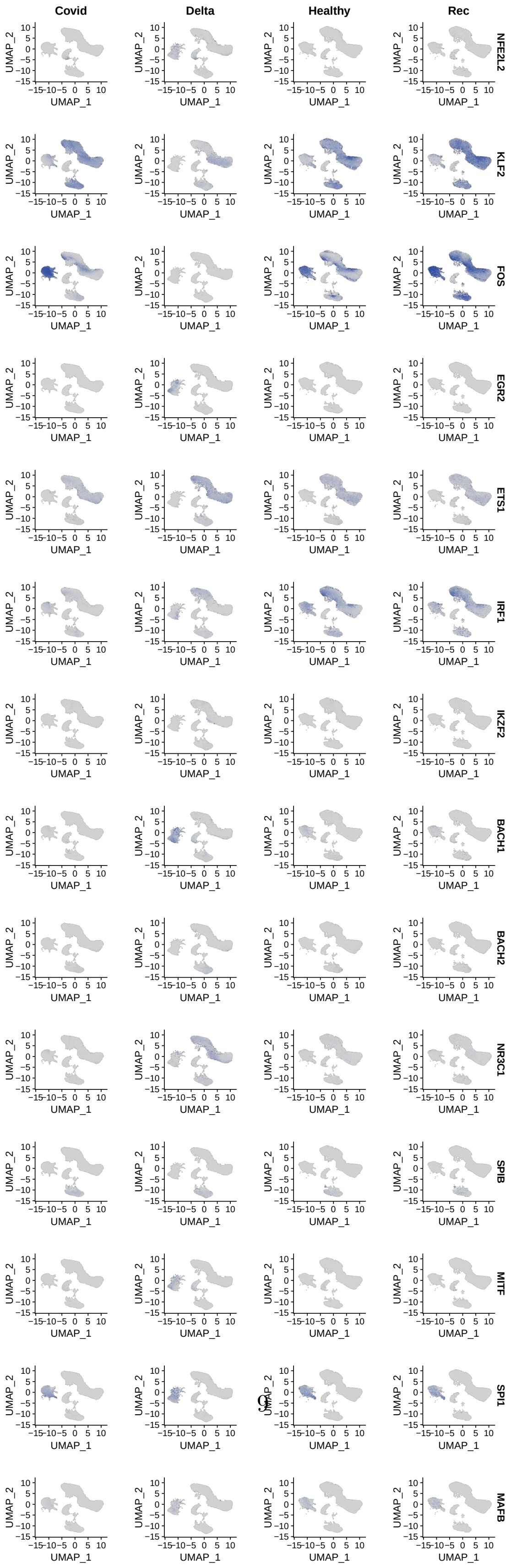

Supplementary figure 9 UMAP with expression of TFs, splitted by cohorts.

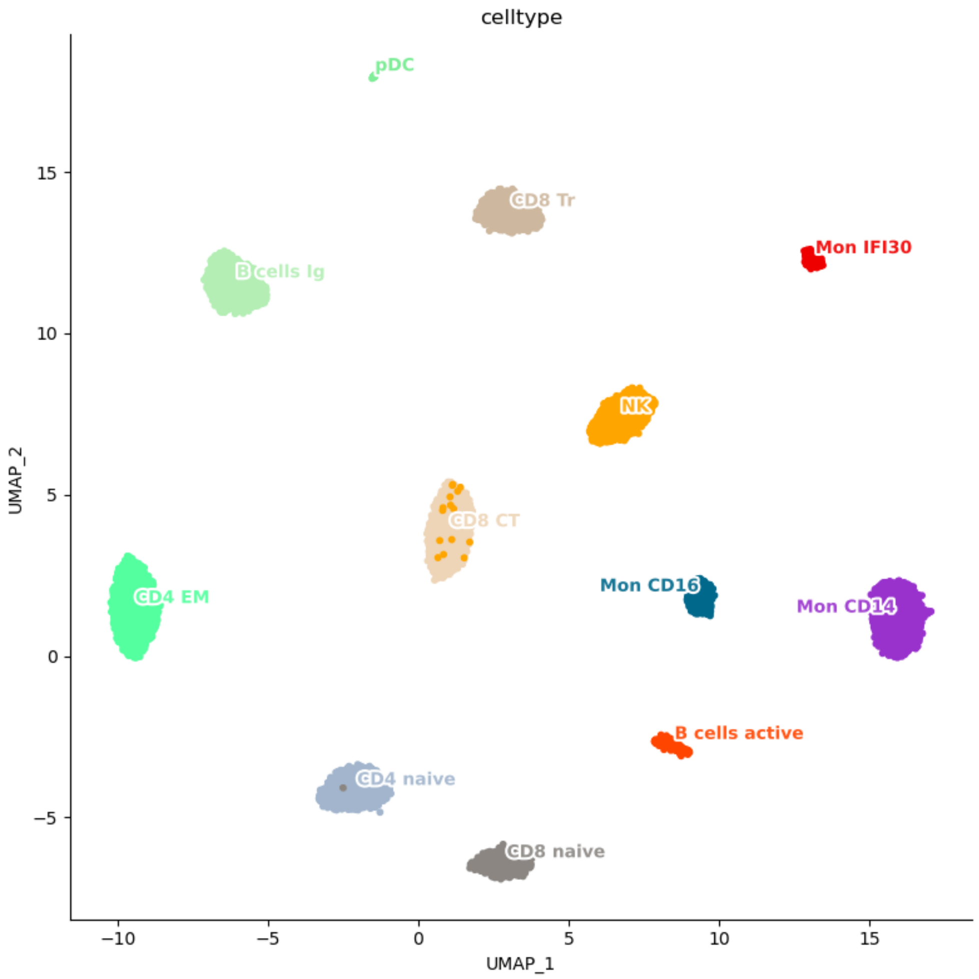

Supplementary figure 10 UMAP result of pseudomultiomics integration of scRNA-seq and scATAC-seq data using the SCENIC+ tool.

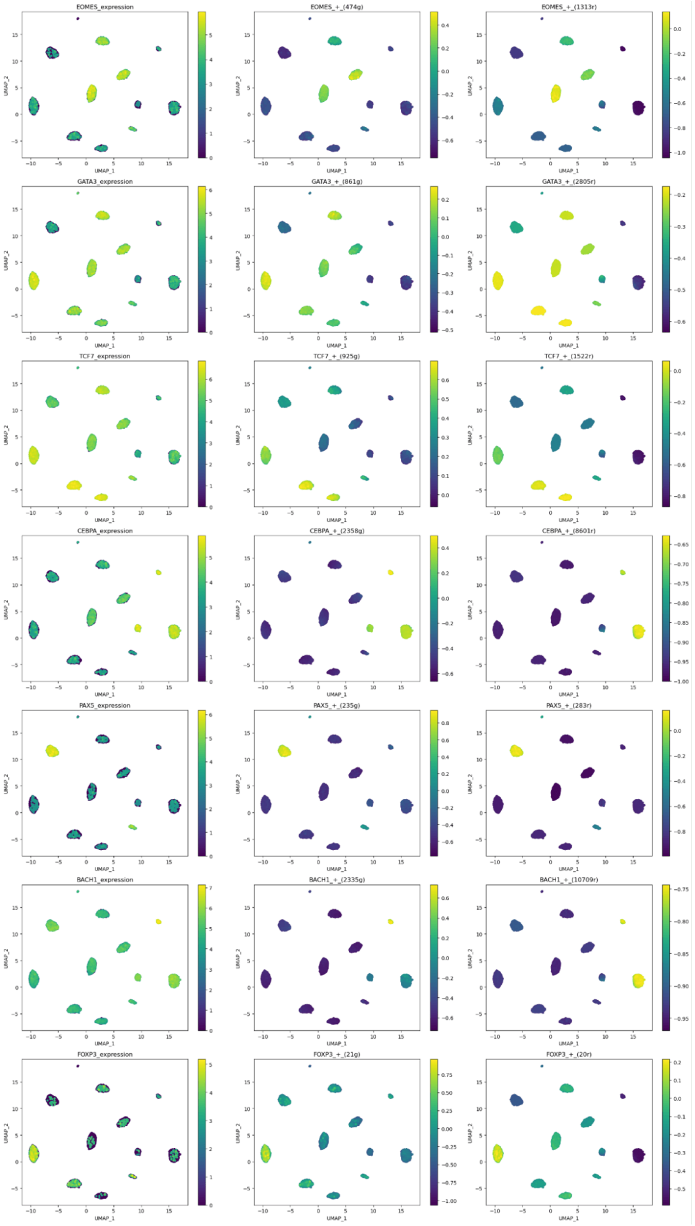

Supplementary figure 11 UMAP of the result of pseudomultiomics integration of scRNA-seq and scATAC-seq data using the SCENIC+ tool with highlighting of key regulons.

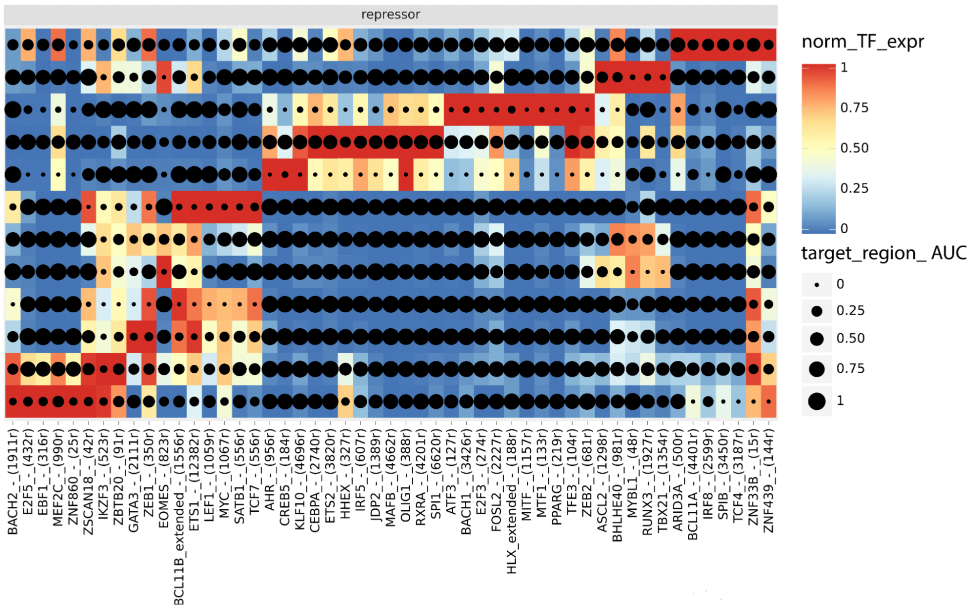

Supplementary figure 12 Heatmap/dotplot with TFs expression of repressing regulons across cell types.

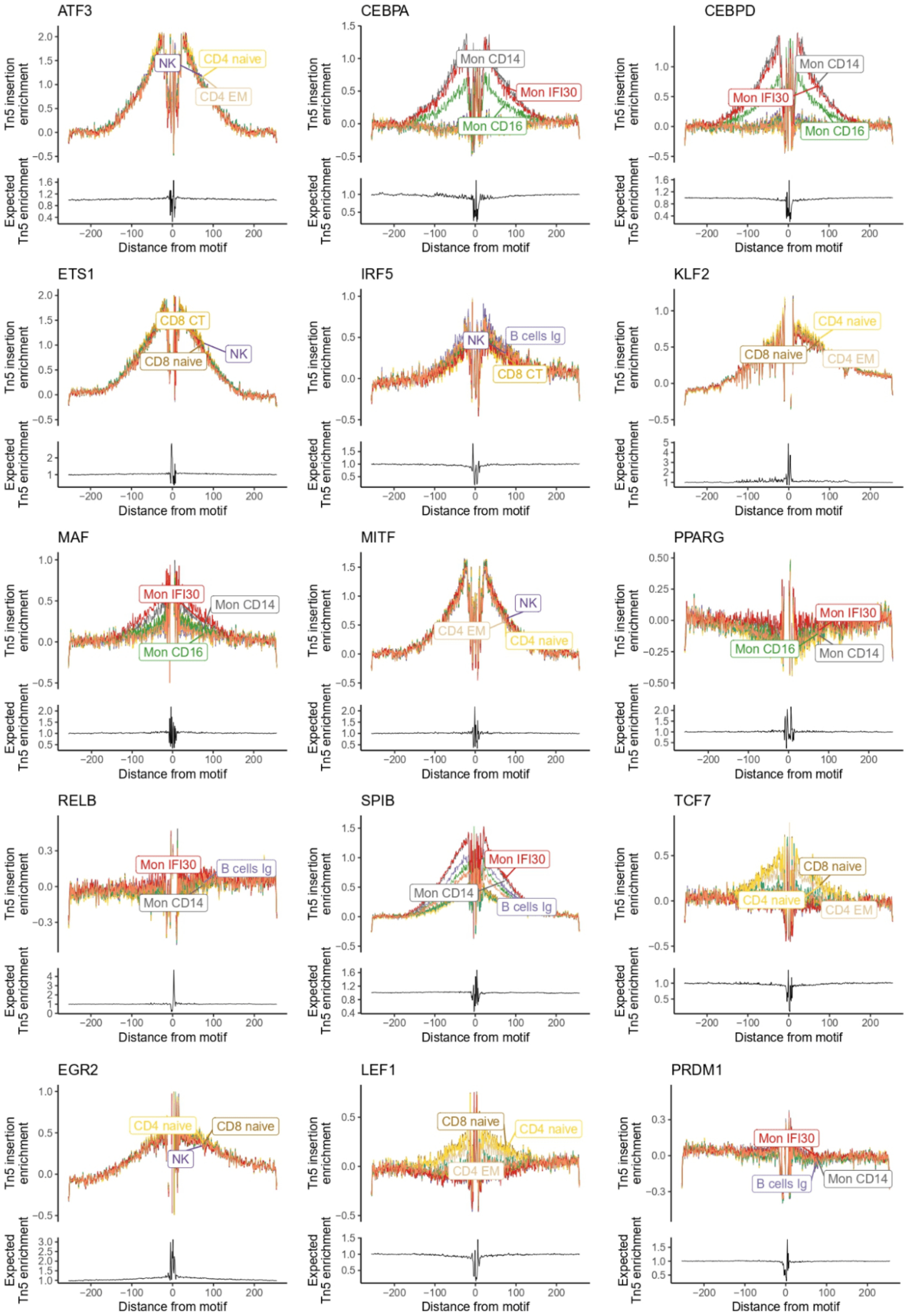

Supplementary figure 13 Footprint plots for key transcription factors between cohorts.

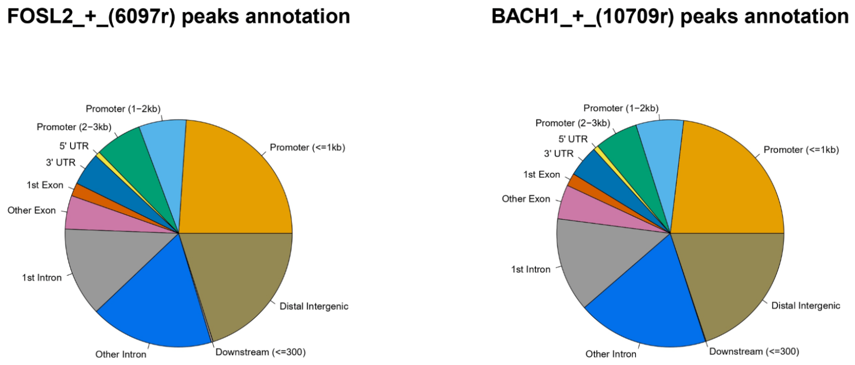

Supplementary figure 14 Annotation of peaks upregulated by FOSL2 and BACH1.

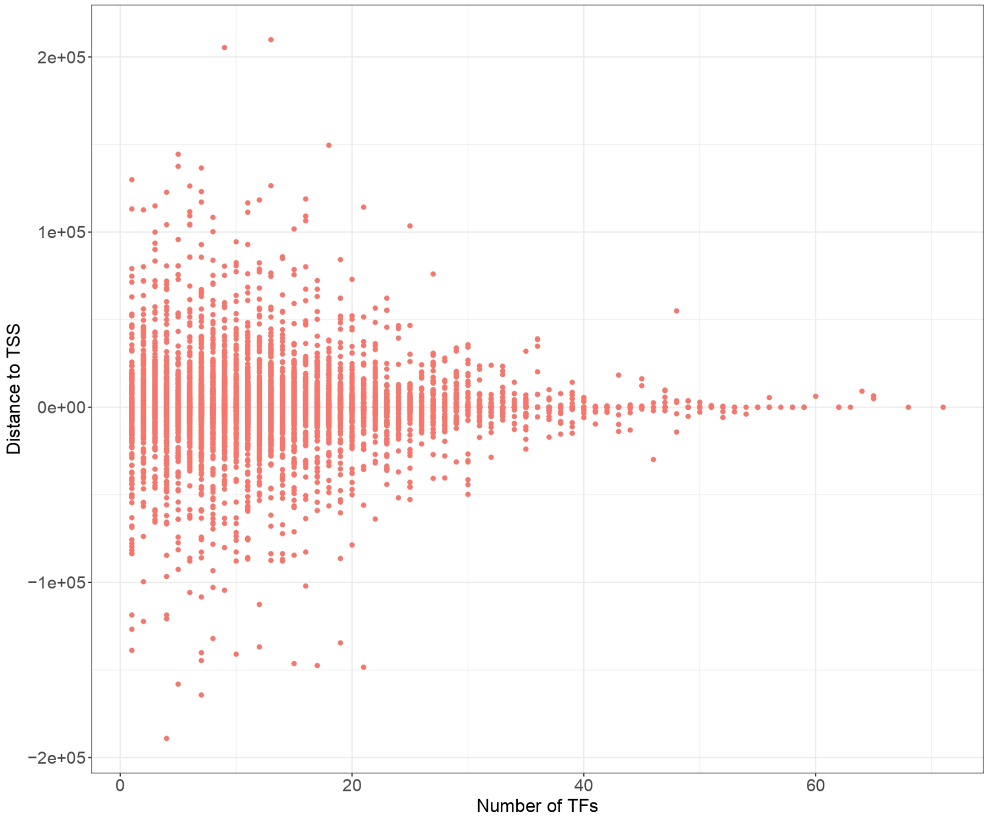

Supplementary figure 15 Scatterplot of peak distance to TSS vs number of TFs regulating a peak.

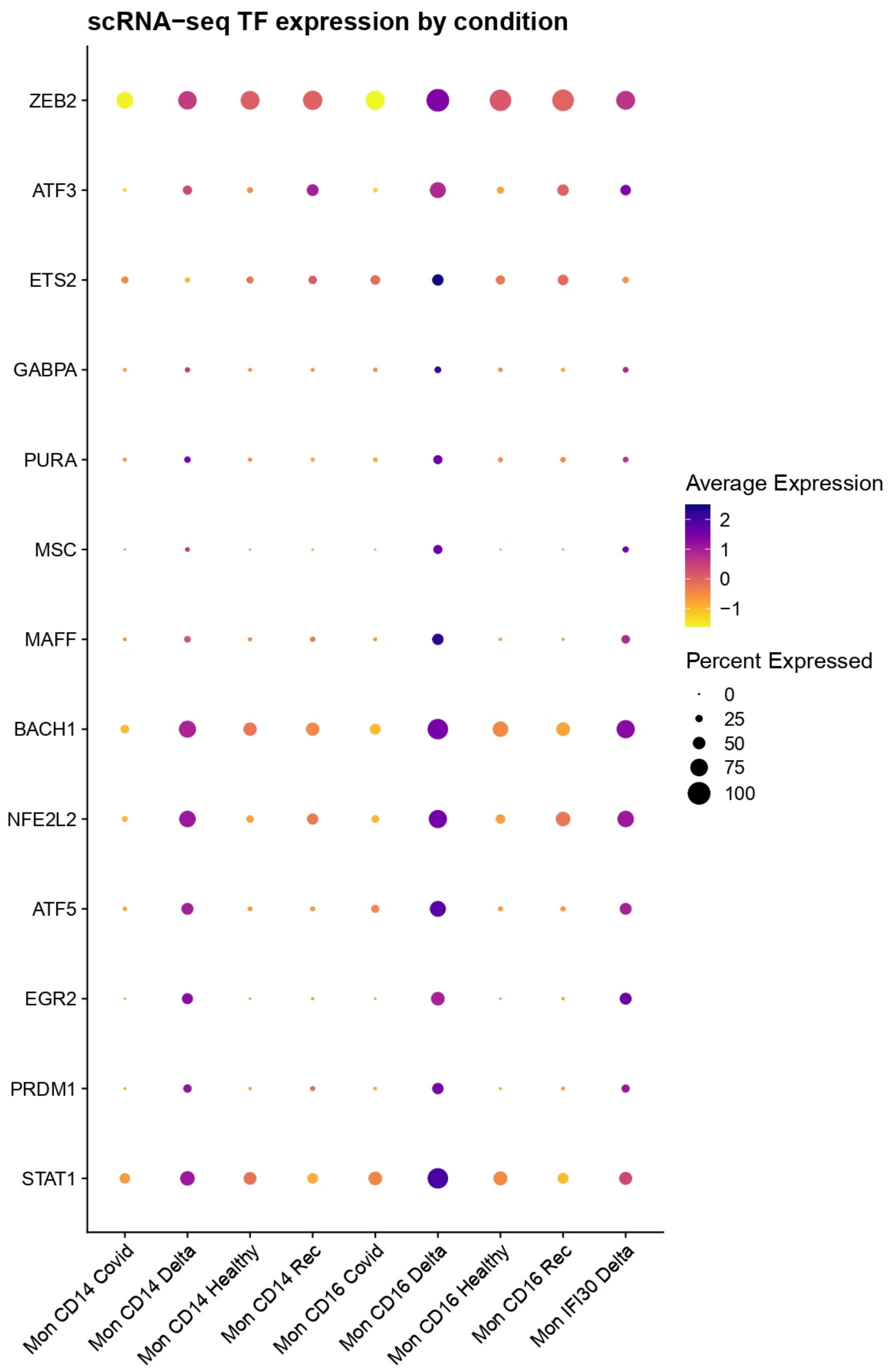

Supplementary figure 16 Dotplot of expression of key transcription factors in different monocyte subtypes between cohorts.

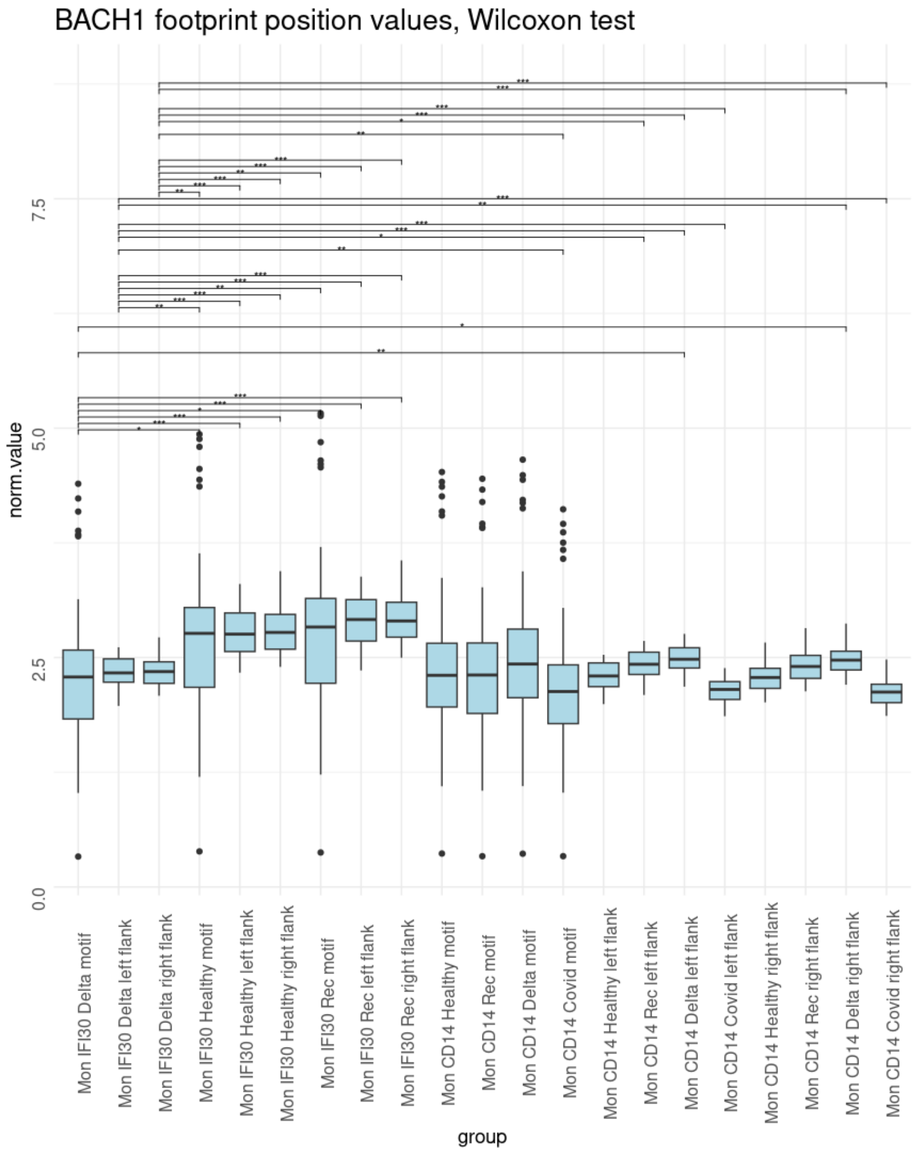

Supplementary figure 17 Pairwise Wilcoxon test comparison for BACH1 footprint Tn5 insertion scores of the motif itself (20 positions), and its left and right flanking regions (20 positions for every flank).

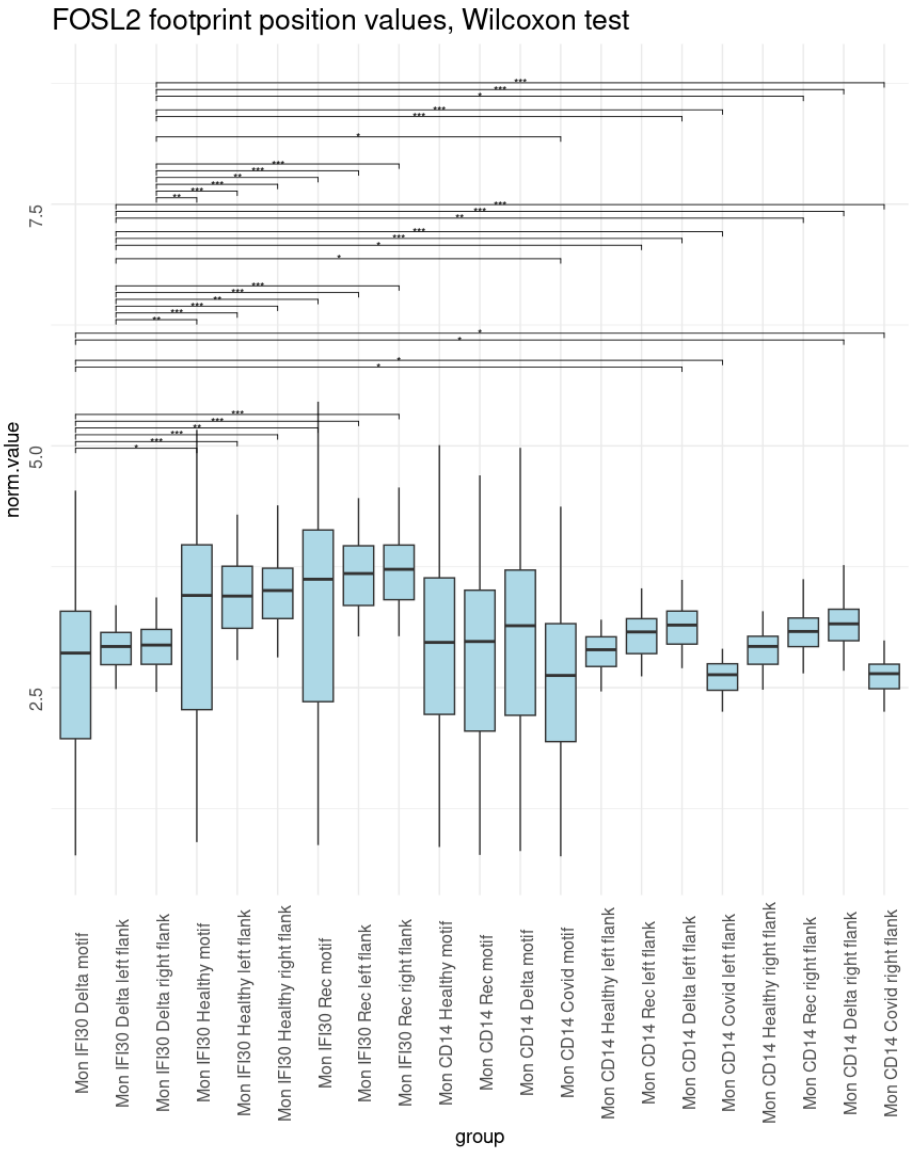

Supplementary figure 18 Pairwise Wilcoxon test comparison for FOSL2 footprint Tn5 insertion scores of the motif itself (20 positions), and its left and right flanking regions (20 positions for every flank).

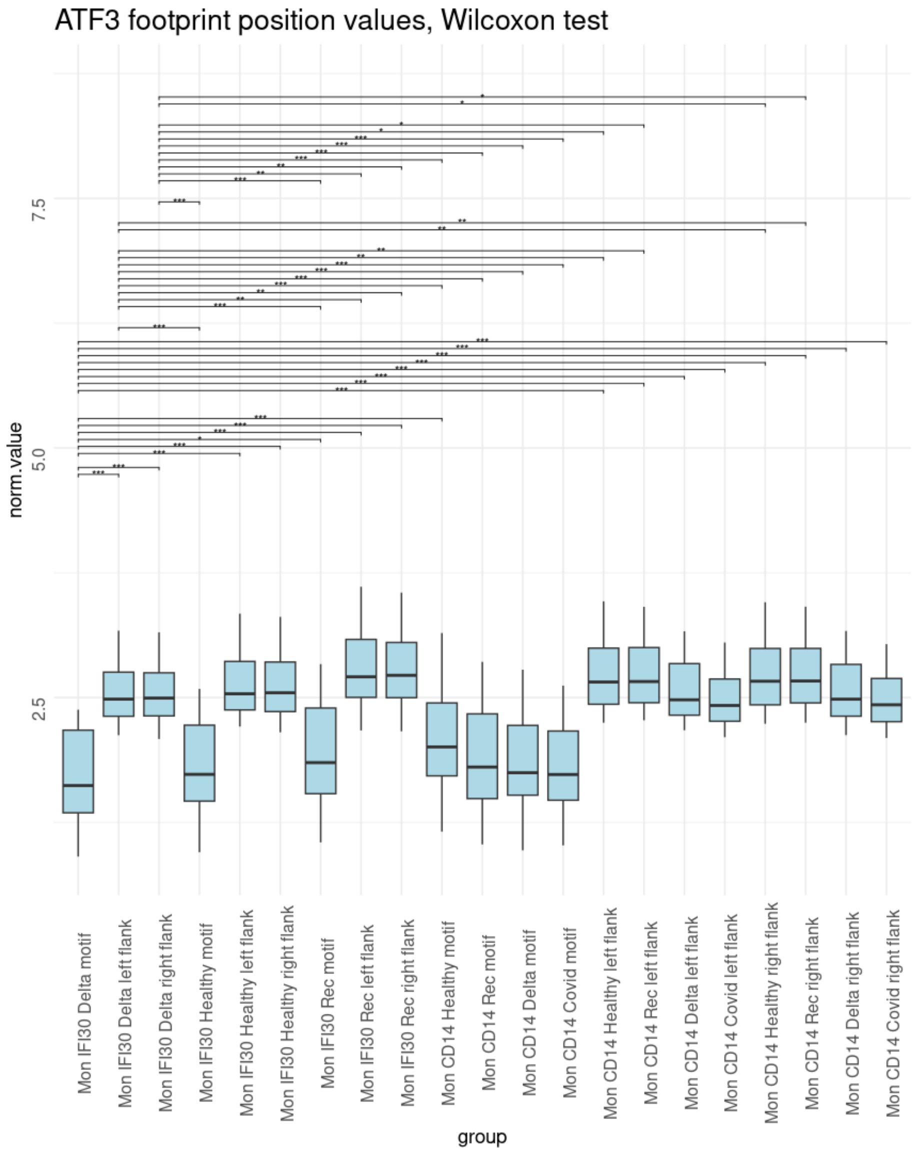

Supplementary figure 19 Pairwise Wilcoxon test comparison for ATF3 footprint Tn5 insertion scores of the motif itself (20 positions), and its left and right flanking regions (20 positions for every flank).

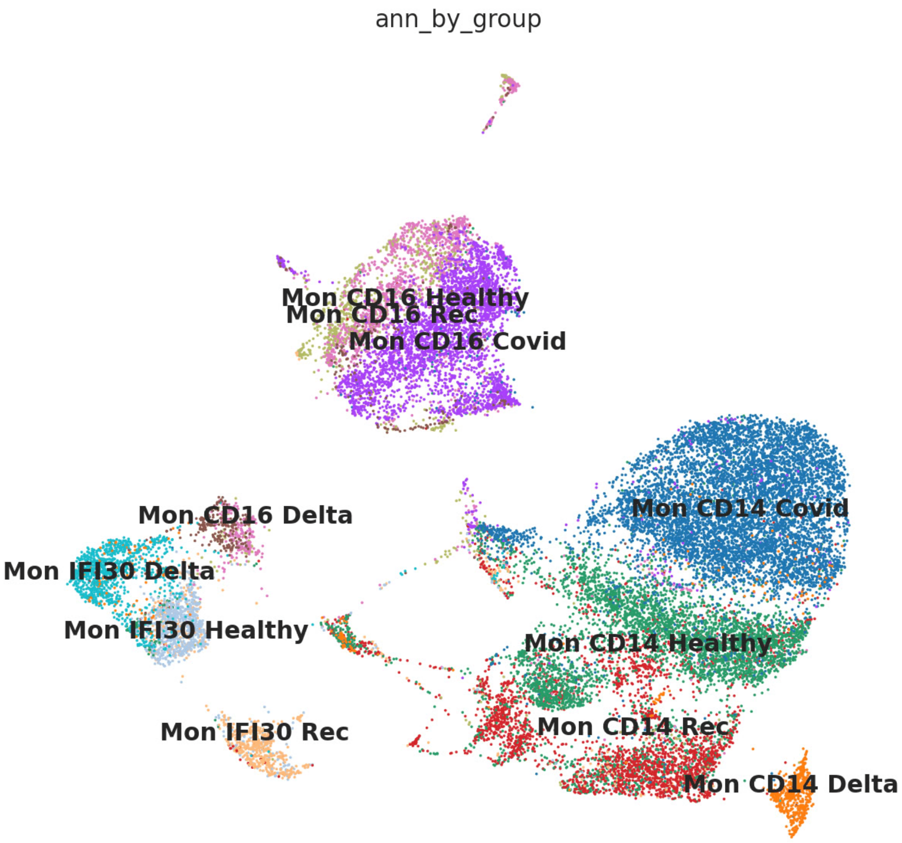

Supplementary figure 20 UMAP integration of monocytes obtained using PeakVI neural network.

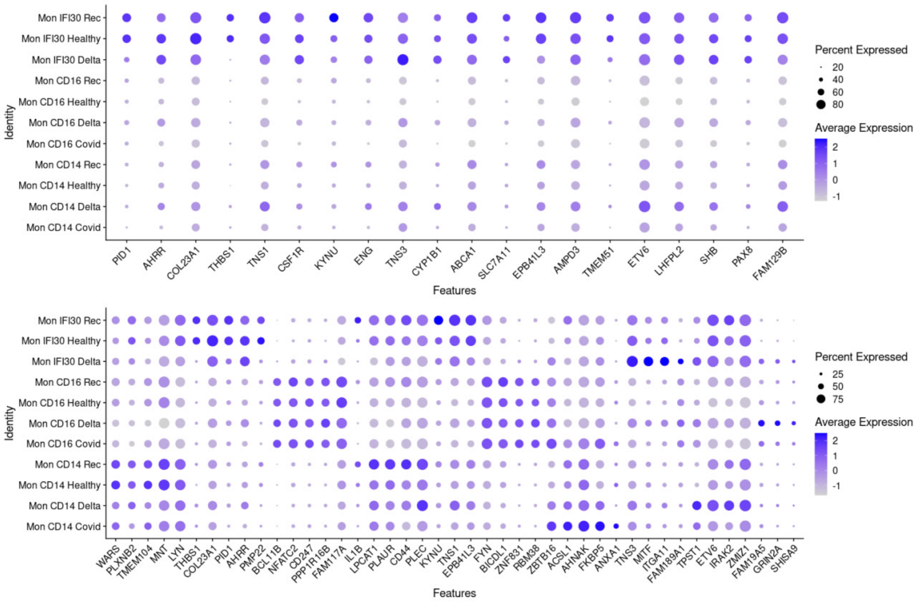

Supplementary figure 21 Genes with differential activity identified across monocytes and cohorts based on the scATAC-seq signal.

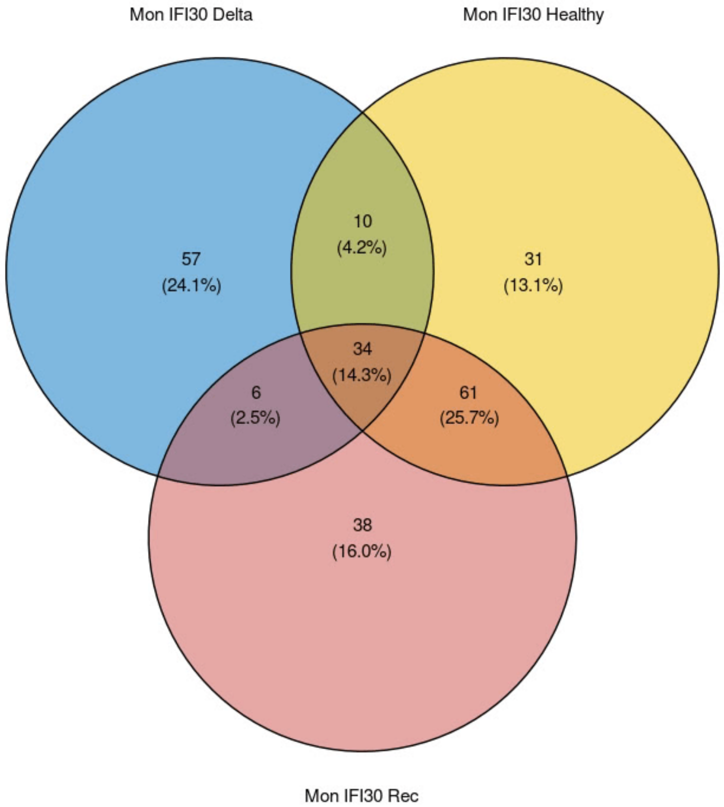

Supplementary figure 22 Venn diagram with overlap of the cohort specific Mon IFI30 marker genes (estimated via gene activity approximation) with Mon IFI30 marker genes obtained with scRNA-seq data.

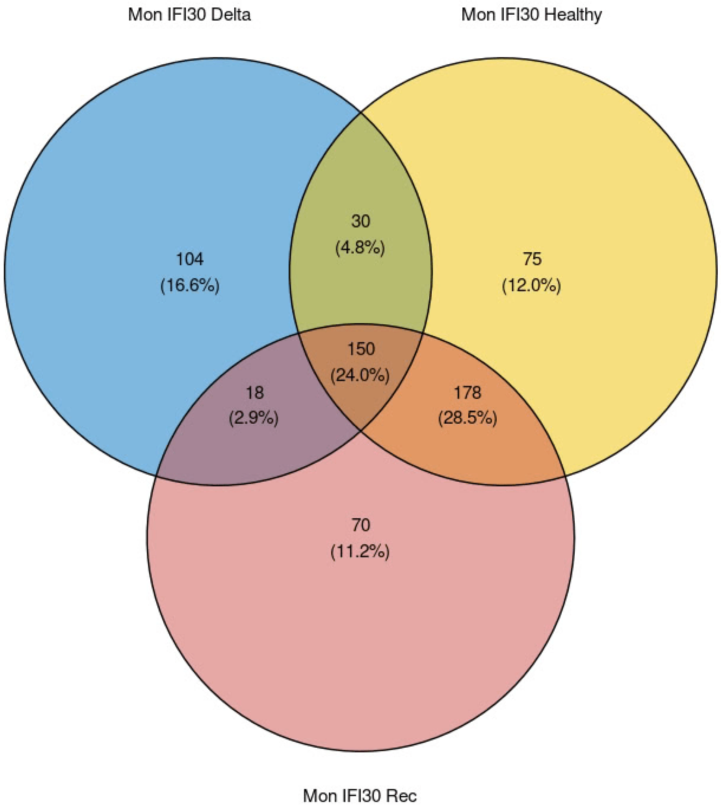

Supplementary figure 23 Intersection of the cohort specific Mon IFI30 peaks with general Mon IFI30 marker peaks.

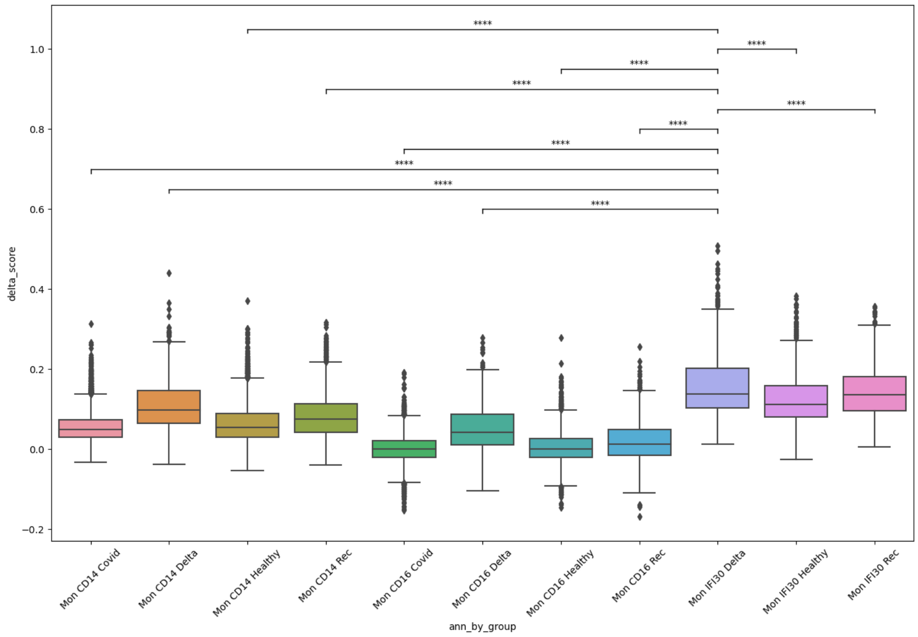

Supplementary figure 24 Boxplots with module scores estimated based on the approximated gene activity from chromatin accessibility data. Mon IFI30 marker genes obtained with scRNA-seq have been used as input gene module.

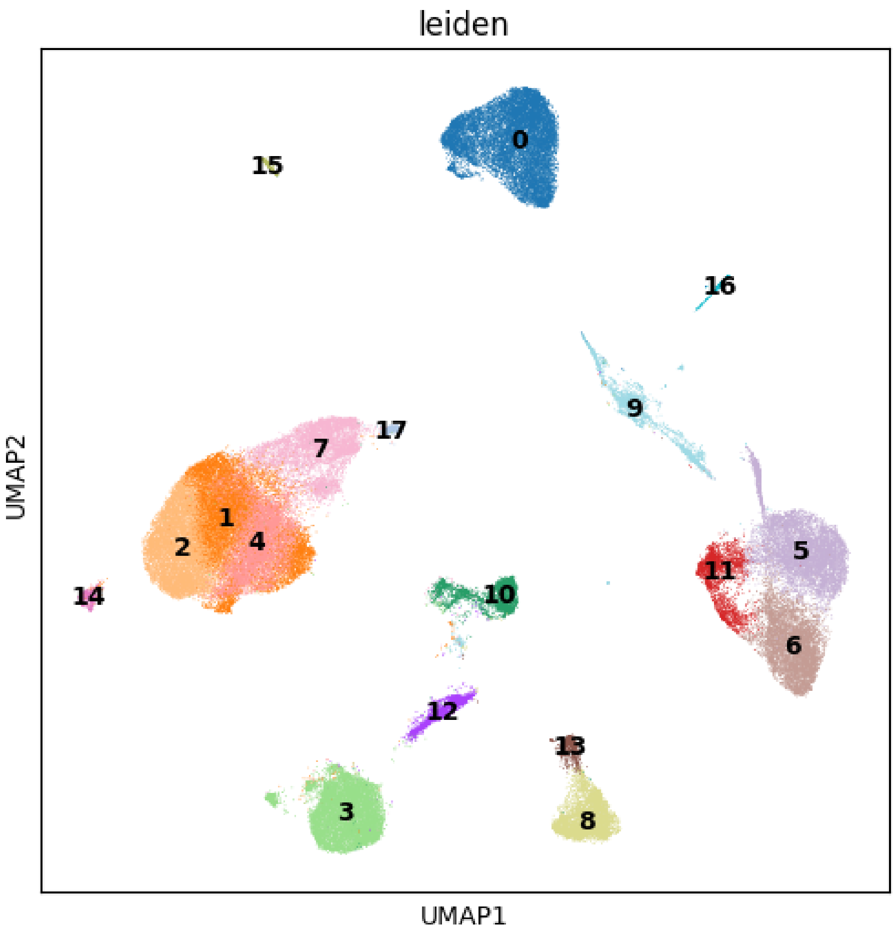

Supplementary figure 25 UMAP integration of public scATAC-seq PBMC from Wimmers F et al. obtained using SnapATAC2 package.

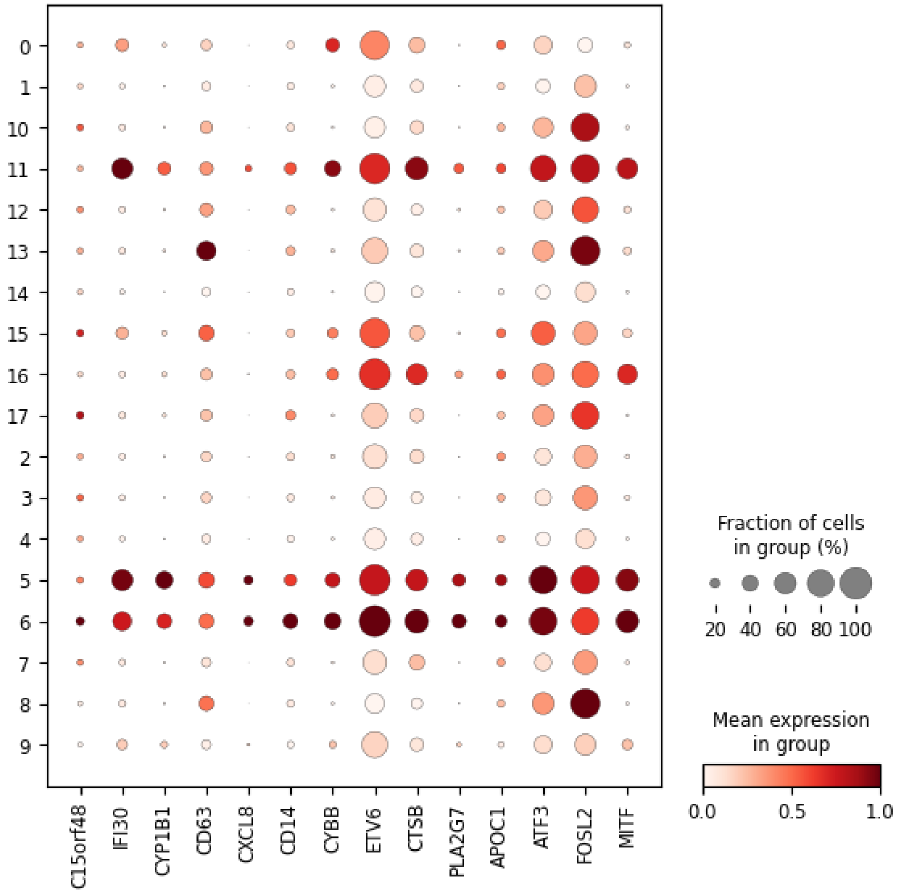

Supplementary figure 26 PBMC myeloid cells from infants with acute Omicron COVID-19 and pre-Omicron demonstrate elevated chromatin accessibility of Mon IFI30 marker genes.

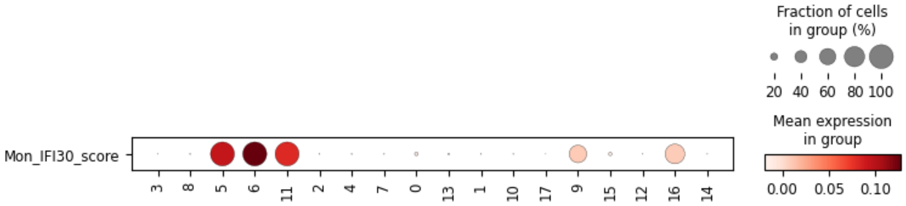

Supplementary figure 27 Dotplot for Mon IFI30 marker peaks module score for scATAC-seq clusters from Wimmers F et al .

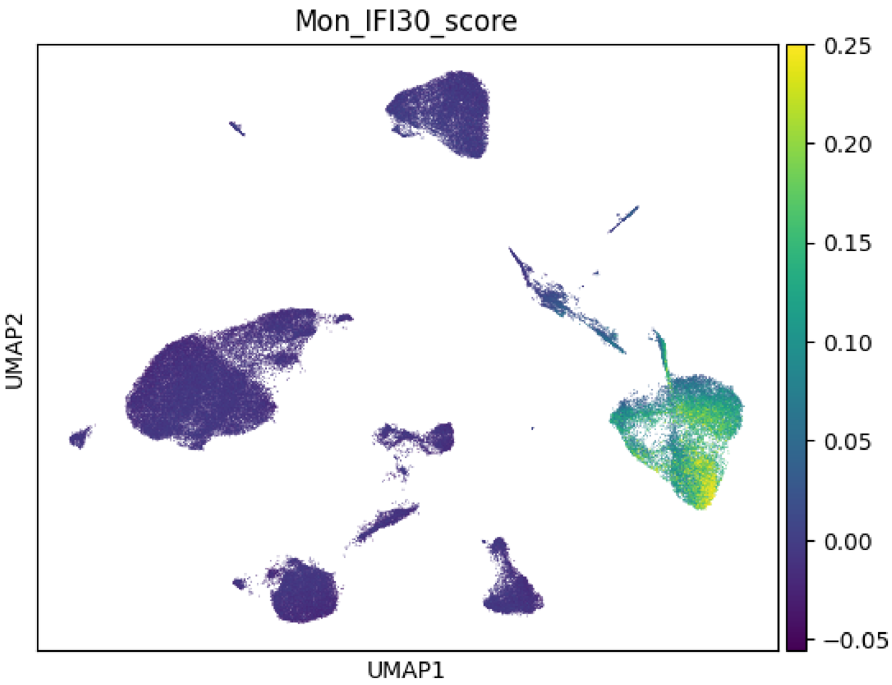

Supplementary figure 28 Feature plot with module score highlighting activity of the Mon IFI30 marker peaks in scATAC-seq data from Wimmers F et al.

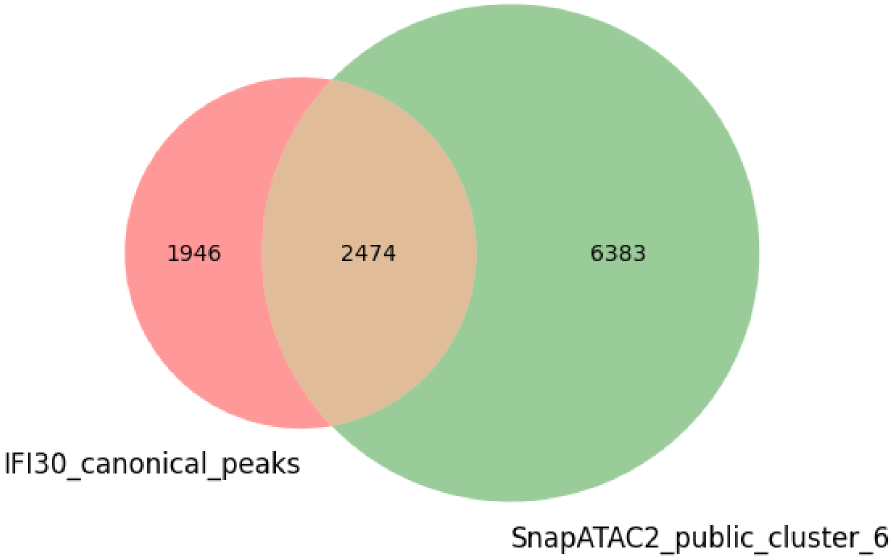

Supplementary figure 29 Venn diagram of Mon IFI30 and cluster 6 marker peaks (scATAC-seq data from Wimmers F et al.)

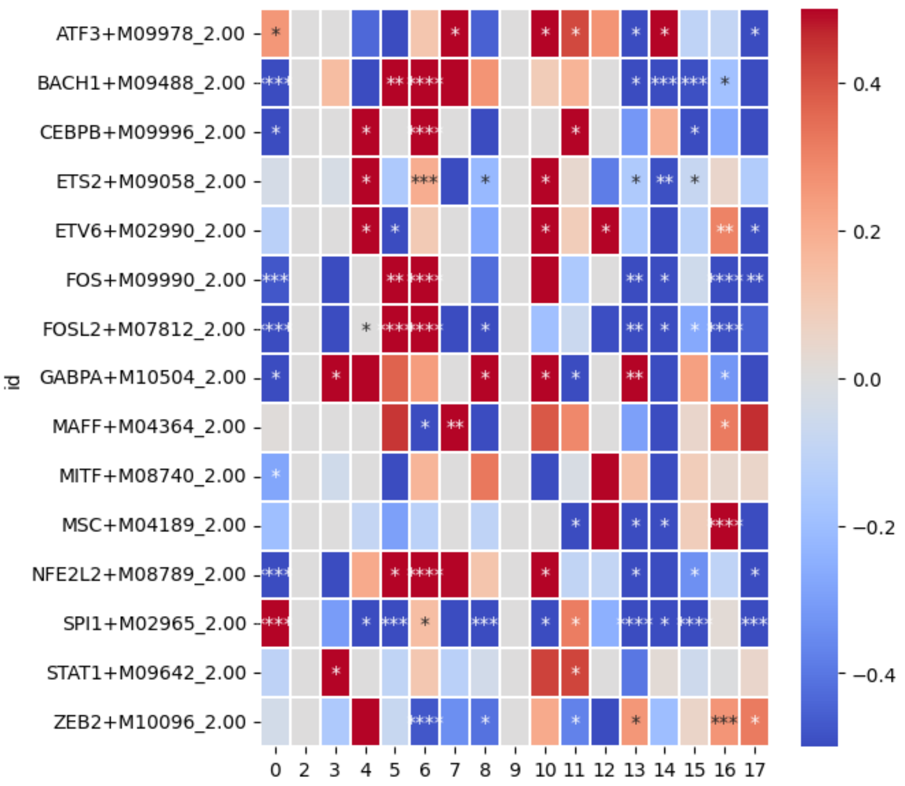

Supplementary figure 30 Heatmap with differential enriched TF motifs for cell clusters in the scATAC-seq from Wimmers F et al. P-values calculated with the Binomial test (1 vs rest) and shown with stars (* for p-value <= 0.05, ** for p-value <= 0.01, *** for p-value <= 0.001, **** for p-value <= 0.0001).

Table 1 Description of the datasets used in the study

SampleID	Severity	Dataset	Experiment
67	Mild/Moderate	Covid	scATAC-seq/scRNA-seq
68	Mild/Moderate	Covid	scATAC-seq/scRNA-seq
69	Mild/Moderate	Covid	scATAC-seq/scRNA-seq
108	Reconvalescence	Rec	scATAC-seq/scRNA-seq
109	Healthy	Healthy	scATAC-seq/scRNA-seq
110	Healthy	Healthy	scATAC-seq/scRNA-seq
111	Healthy	Healthy	scATAC-seq
112	Healthy	Healthy	scRNA-seq/scRNA-seq
113	Healthy	Healthy	scATAC-seq/scRNA-seq
114	Healthy	Healthy	scATAC-seq/scRNA-seq
115	Healthy	Healthy	scATAC-seq/scRNA-seq
134	Mild/Moderate	Covid	scATAC-seq/scRNA-seq
144	Mild/Moderate	Covid	scATAC-seq/scRNA-seq
147	Mild/Moderate	Covid	scATAC-seq/scRNA-seq
148	Mild/Moderate	Covid	scATAC-seq/scRNA-seq
151	Mild/Moderate	Covid	scATAC-seq/scRNA-seq
165	Healthy	Healthy	scATAC-seq/scRNA-seq
166	Healthy	Healthy	scATAC-seq/scRNA-seq
167	Reconvalescence	Rec	scATAC-seq/scRNA-seq
170	Healthy	Healthy	scATAC-seq
172	Reconvalescence	Rec	scATAC-seq/scRNA-seq
207	Healthy	Healthy	scATAC-seq/scRNA-seq
208	Reconvalescence	Rec	scATAC-seq/scRNA-seq
210	Reconvalescence	Rec	scATAC-seq
212	Healthy	Healthy	scATAC-seq
215	Healthy	Healthy	scATAC-seq
218	Reconvalescence	Rec	scATAC-seq/scRNA-seq
245	Reconvalescence	Rec	scATAC-seq/scRNA-seq
413	Mild/Moderate	Covid	scATAC-seq/scRNA-seq
414	Mild/Moderate	Covid	scATAC-seq/scRNA-seq
415	Mild/Moderate	Covid	scATAC-seq/scRNA-seq
416	Mild/Moderate	Covid	scATAC-seq/scRNA-seq
417	Mild/Moderate	Covid	scATAC-seq/scRNA-seq
418	Mild/Moderate	Covid	scATAC-seq/scRNA-seq
419	Mild/Moderate	Covid	scATAC-seq/scRNA-seq
420	Mild/Moderate	Covid	scRNA-seq
561	Severe/Critical	Delta	scRNA-seq
562	Severe/Critical	Delta	scATAC-seq/scRNA-seq
563	Severe/Critical	Delta	scATAC-seq/scRNA-seq
564	Severe/Critical	Delta	scATAC-seq/scRNA-seq
565	Severe/Critical	Delta	scRNA-seq
566	Severe/Critical	Delta	scRNA-seq
567	Severe/Critical	Delta	scATAC-seq/scRNA-seq
568	Severe/Critical	Delta	scRNA-seq
569	Severe/Critical	Delta	scRNA-seq
573	Severe/Critical	Delta	scRNA-seq
574	Severe/Critical	Delta	scATAC-seq/scRNA-seq
